# Competition-based screening helps to secure the evolutionary stability of a defensive microbiome

**DOI:** 10.1186/s12915-021-01142-w

**Published:** 2021-09-15

**Authors:** Sarah F. Worsley, Tabitha M. Innocent, Neil A. Holmes, Mahmoud M. Al-Bassam, Morten Schiøtt, Barrie Wilkinson, J. Colin Murrell, Jacobus J. Boomsma, Douglas W. Yu, Matthew I. Hutchings

**Affiliations:** 1 School of Biological Sciences, Norwich Research Park, University of East Anglia, Norwich, Norfolk, NR4 7TJ UK; 2 Centre for Social Evolution, Section for Ecology and Evolution, Department of Biology, University of Copenhagen, Copenhagen, Denmark; 3 Department of Molecular Microbiology, John Innes Centre, Norwich Research Park, Norwich, Norfolk, NR4 7UH UK; 4 School of Environmental Sciences, Norwich Research Park, University of East Anglia, Norwich, Norfolk, NR4 7TJ UK; 5 State Key Laboratory of Genetic Resources and Evolution, Kunming Institute of Zoology, Chinese Academy of Sciences, Kunming, 650223 Yunnan China; 6 Center for Excellence in Animal Evolution and Genetics, Chinese Academy of Sciences, Kunming, 650223 Yunnan China

**Keywords:** Antibiotics, Attini, Game theory, Defensive microbiome, Mutualism, Actinobacteria, Partner, Leaf-cutting ants, *Pseudonocardia*, Interference competition, Horizontal acquisition, Symbiosis

## Abstract

**Background:**

The cuticular microbiomes of *Acromyrmex* leaf-cutting ants pose a conundrum in microbiome biology because they are freely colonisable, and yet the prevalence of the vertically transmitted bacteria *Pseudonocardia*, which contributes to the control of *Escovopsis* fungus garden disease, is never compromised by the secondary acquisition of other bacterial strains. Game theory suggests that *competition-based screening* can allow the selective recruitment of antibiotic-producing bacteria from the environment, by providing abundant resources to foment interference competition between bacterial species and by using *Pseudonocardia* to bias the outcome of competition in favour of antibiotic producers.

**Results:**

Here, we use RNA-stable isotope probing (RNA-SIP) to confirm that *Acromyrmex* ants can maintain a range of microbial symbionts on their cuticle by supplying public resources. We then used RNA sequencing, bioassays, and competition experiments to show that vertically transmitted *Pseudonocardia* strains produce antibacterials that differentially reduce the growth rates of other microbes, ultimately biassing the bacterial competition to allow the selective establishment of secondary antibiotic-producing strains while excluding non-antibiotic-producing strains that would parasitise the symbiosis.

**Conclusions:**

Our findings are consistent with the hypothesis that competition-based screening is a plausible mechanism for maintaining the integrity of the co-adapted mutualism between the leaf-cutting ant farming symbiosis and its defensive microbiome. Our results have broader implications for explaining the stability of other complex symbioses involving horizontal acquisition.

**Supplementary Information:**

The online version contains supplementary material available at 10.1186/s12915-021-01142-w.

## Background

The diversity of insect-associated microbial communities is staggering. They may consist of single intracellular symbionts with reduced genomes owing to coadaptation at one extreme [1] and to dynamic microbiomes in open host compartments such as guts at the other end of the scale [2]. Insect microbiomes have been intensively studied across a range of species, and there is increasing consensus regarding their vital contributions to host fitness throughout ontogenetic development [3–6]. However, the stability and cooperative characteristics of complex microbiomes are a paradox. While relentless competition is the default setting of the microbial world [7], hosts appear to evolve control by holding their microbiome ecosystems on a leash [8], but how dynamic stability under continuing turnover is achieved remains unclear. Despite an abundance of microbiome research, recent reviews have concluded that “integration between theory and experiments is a crucial ‘missing link’ in current microbial ecology” [9] and that ‘our ability to make predictions about these dynamic, highly complex communities is limited’ [10].

Game theory suggests a compelling solution to the unity-in-diversity paradox by showing that *competition-based screening* can be a powerful mechanism to maintain cooperative stability. Screening is likely to work when hosts evolve (1) to provide nutrients and/or space to foment competition amongst symbionts, thus creating an attractive but ‘demanding’ environment, and (2) to skew the resulting competition such that mutualistic symbionts enjoy a competitive advantage. Competitive exclusion then ‘screens in’ mutualists and ‘screens out’ parasitic and free-rider symbionts [11–13]. Screening is conceptually clearest when the symbiont trait that confers competitive superiority is the same as (or strongly correlated with) the trait that benefits the host. An illustration of such correlated functionality was provided by Heil [14] who showed that ant-hosting acacia plants provide copious food bodies, which fuels the production of numerous, actively patrolling ant workers. The ant species whose colony invests in greater numbers of aggressive workers outcompetes other ant colonies trying to establish on the same plant, and the same investment in aggressive workers is likely to better protect the host plants against herbivores [14].

Screening has also been suggested to act in animal-microbe symbioses. For instance, Tragust et al. [15] showed that carpenter ants acidify their own stomachs by swallowing acidopore secretions. Entomopathogenic bacteria are then rapidly killed off, whereas the co-adapted gut bacterial symbiont *Asaia* sp. (Acetobacteraceae) exhibits a lower mortality rate and maintains itself in the midgut. Addressing a similar question, Itoh et al. [16] used co-inoculation experiments to show that environmentally recruited but co-adapted ‘native’ *Burkholderia* symbionts outcompete non-native bacteria in the gut of their bean bug host, even though they are able to establish in the absence of the ‘native’ symbiont. Finally, Ranger et al. [17] showed that ambrosia beetles selectively colonise physiologically stressed trees, which have a high ethanol titre due to anaerobic respiration. The vertically transmitted fungal symbionts of these beetles have evolved to detoxify the ethanol whereas competing weedy fungi remain inhibited.

Competition-based screening seems particularly apt for the establishment of protective microbiomes [12, 13] because although the production of bioactive compounds such as antibiotics is highly complex, leading to a wide diversity of chemical structures and resistance mechanisms, natural selection is expected to reinforce the correlation between the traits of antibiotic production and antibiotic resistance, since production without resistance by the same cell would be suicidal. Scheuring and Yu [12] thus proposed that if a host species provides copious food resources and if non-producer strains have the faster growth rate, the microbiome becomes ‘bistable’, meaning that two equilibrium outcomes are possible. If the non-producers start with a higher initial abundance, their faster growth rate allows them to take over the host space and exclude the antibiotic-producers before the latter can produce high concentrations of antibiotic, whereas if the slower-growing antibiotic producers start with a higher initial abundance, they have a large enough population to produce enough antibiotic to kill off non-producers and grow to take over the host space. In this light, vertical transmission of an antibiotic producer strain by the host can ensure that the microbiome always starts with a higher abundance of antibiotic producers.

The conservative assumption in this argument is that the non-producers are given the faster growth rate, which is likely, since they do not pay the cost of antibiotic production and since non-producers are more common than producers in the environment, which implies that in any random sample of microbial colonisers from the environment, at least some of the non-producers are likely to have faster growth rates than producers. Under such circumstances, vertical transmission of a primary antibiotic producer can result in the selective horizontal acquisition (recruitment) of additional, antibiotic-producing bacteria from the environment, because antibiotic-producers should be superior competitors in food- and antibiotic-filled environments. The resulting, more diverse, microbiomes are then largely purged of free-riding non-beneficial strains [8, 11, 12]. Previous research has shown that the protective, cuticular microbiome of *Acromyrmex echinatior* leaf-cutting ants (Formicidae, Attini) is an ideal model system to test whether screening can act as a leash in the ecosystem-on-a-leash perspective [8, 18].

*Acromyrmex* worker ants forage for fresh leaf fragments to provision their co-evolved fungus garden mutualist *Leucoagaricus gongylophorus* [19, 20]. The fungal cultivar produces gongylidia, nutrient-rich swellings that are the sole food source for the queen and larvae [21, 22] and the predominant food source for the workers who also ingest plant sap and fruit juice in addition to fungal food [23]. However, *Leucoagaricus* is at risk of being parasitised by the specialised, coevolved mould *Escovopsis weberi*, which can degrade the fungal cultivar and also cause severe ant paralysis and mortality [24–27]. To prevent infections, leaf-cutting ants have evolved a range of weeding and grooming behaviours [28–30], and *A. echinatior* and other *Acromyrmex* species also maintain filamentous actinomycete bacteria that grow as a white bloom on the cuticles of large workers. These bacteria produce antimicrobials that inhibit the growth of *E. weberi* [24, 26, 31]. In Panama, where almost all fieldwork on this multipartite symbiosis has been carried out, the cuticle of *Acromyrmex* workers is dominated by one of two vertically transmitted strains of *Pseudonocardia*, named *P. octospinosus* (Ps1) and *P. echinatior* (Ps2) [18, 32, 33].

Newly eclosed large workers are inoculated with the vertically transmitted *Pseudonocardia* strain by their nestmates. This blooms over the cuticle, reaching maximum coverage after ca. 6 weeks, before shrinking back to the propleural plates as the ants mature to assume foraging tasks [34, 35]. However, several studies have also identified other actinomycete strains on the propleural plates of *A. echinatior*, which are presumed to be acquired from the environment [18, 36–40]. This includes species of the bacterial genus *Streptomyces*, which have been identified on ants maintained in laboratory-based colonies [36–38], as well as in a 16S rRNA gene amplicon sequencing study of ants sampled from their native environment, although many of these could also have been close relatives of *Streptomyces* [18]. Species of this actinomycete genus produce a variety of antimicrobials so their additional presence may suggest a form of multi-drug therapy against *Escovopsis* [37–39]. However, these putative functions remain enigmatic because *Streptomyces* symbionts were never found on the callow workers [18] that execute hygienic and defensive fungus garden tasks [35]. In terms of resources, the propleural plates have a high concentration of tiny subcuticular glands, which are presumed to supply the cuticular microbiome with resources [41]. These plates can thus be conjectured to create the food-rich but antibiotic-laden demanding environment that competition-based screening assumes, because the vertically transmitted native *Pseudonocardia* symbiont always colonises the propleural plates first [12, 35]. We have previously shown that both *P. octospinosus* and *P. echinatior* encode and make antibacterial compounds that inhibit multiple unicellular bacteria but do not inhibit *Streptomyces* species [32]. However, the other key elements of the screening hypothesis have remained untested.

The present study carries out a series of tests of competition-based screening, using the cuticular microbiome of *A. echinatior* as an open symbiotic ecosystem where the host nonetheless holds the leash, by controlling resource provisioning rates and by having first inoculated workers with *Pseudonocardia*. The co-adapted defensive *Pseudonocardia* symbiont, which is advantaged due to the priority effects associated with vertical transmission, is then expected to skew subsequent competition amongst an unspecified number of symbionts randomly colonising from the environment. We used RNA-SIP (stable isotope probing) to show that the ants provide a food resource on their cuticles that is consumed by multiple bacterial species, thus showing both that the resource is public and that multiple bacterial species can become established in the cuticular microbiome. We then use RNA sequencing to show, in vivo, that mutualistic *P. octospinosus* and *P. echinatior* strains express antibacterial biosynthetic gene clusters (BGCs) on the ant cuticle. Next, we show that diffusible metabolites of these *Pseudonocardia* species exhibit broad-spectrum antibacterial activity in vitro, but only weakly inhibit *Streptomyces* species isolated from the cuticular microbiome, which we separately and directly show are resistant to a range of antibiotics. Finally, we demonstrate that these elements result in biassed competition, by using in vitro competition experiments to show that slower-growing *Streptomyces* species can competitively exclude faster-growing non-antibacterial-producing species, but only when grown on media infused with *Pseudonocardia* metabolites.

## Results

### The host provides public resources to its cuticular microbiome

RNA-SIP tracks the flow of heavy isotopes from the host to the RNA of microbial partners that metabolise host-derived resources [42–44]. Labelled and unlabelled RNA within a sample can be separated via ultracentrifugation and fractionation; these fractions can be used as templates for 16S rRNA gene amplicon sequencing so that the bacterial taxa that do (and do not) use host-supplied resources can be identified [44]. RNA was chosen over DNA in this experiment to increase the chances of labelling over a short time frame; RNA labelling requires active transcription, whereas DNA labelling requires DNA replication. Actinomycetes, in particular, only massively upregulate DNA replication when they sporulate. A shorter period was also desirable as it reduces any cross-feeding between bacterial species, although we note that any resources acquired by cross-feeding count as public in the screening model because they also, eventually, contribute to bacterial growth and metabolism. Three replicate groups of 22 mature worker ants were fed a 20% (w/v) solution of either ^12^C or ^13^C glucose for 10 days, and then propleural plates were dissected out for total RNA extraction (Additional file 1: Fig. S1). Control feeding experiments demonstrated that a fluorescently labelled glucose-water diet was not transferred to the surface of the propleural plate region of the ants during feeding, and the ants were only ever observed to feed using their mandibles, such that their propleural plates never came into direct contact with the liquid diet (Additional file 1: Fig. S2).

**Fig. 1:**
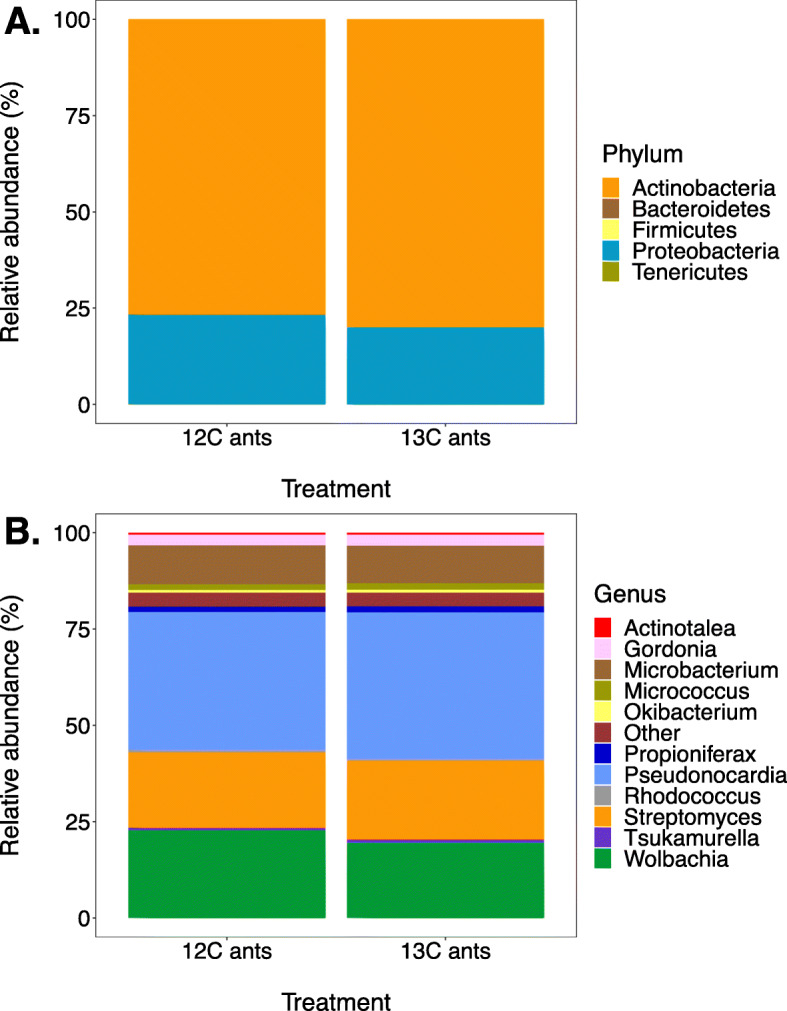
Frequencies of bacteria at different taxonomic levels in unfractionated RNA samples from the propleural plates of ants provided with a 20% (w/v) solution of either ^12^C- or ^13^C-labelled glucose. **A** Phylum resolution, showing that > 75% were Actinobacteria. The remaining reads almost all corresponded to *Wolbachia* (Proteobacteria) so the three other phyla do not appear in the bars (total relative abundance of 0.6% and 0.4% in ^12^C and ^13^C fractions, respectively). As *Wolbachia* is not part of the cuticular microbiome (see text), these results show that the cuticular microbiome is completely dominated by Actinobacteria. **B** Genus-level resolution showing that the cuticular microbiomes are dominated by the native *Pseudonocardia* symbiont followed by appreciable fractions of horizontally acquired *Streptomyces* species and a series of other Actinobacteria at low prevalence

Following cDNA synthesis, 16S rRNA gene amplicon sequencing showed that filamentous actinomycetes dominate the propleural plate samples, making up 76.6% and 78.0% of the total, unfractionated, cDNA samples from ^12^C and ^13^C fed ants, respectively (Fig. 1). The most abundant bacterial genera were *Pseudonocardia*, 35.8% and 38.1% in ^12^C and ^13^C samples, respectively, and *Streptomyces*, 19.7% and 20.5%, respectively. *Wolbachia* made up 22.8% and 19.6%, respectively. *Wolbachia* are known to be associated with the thoracic muscles of *A. echinatior* worker ants where they may have an unspecified mutualistic function [45, 46]. We conclude that these reads came from the trace amount of residual ant tissue on the dissected propleural plates and do not consider them further. More than 95% of the *Pseudonocardia* 16S rRNA gene reads in unfractionated RNA samples were identical in sequence to a single *P. octospinosus* mutualist strain [18, 32]*.* The presence of *Streptomyces* and other actinomycete bacteria is consistent with the interpretation of previous studies that environmentally acquired, antibiotic-producing actinomycetes can establish in the cuticular microbiome [36–40, 47], despite these large workers having first been inoculated with the vertically transmitted symbiont *Pseudonocardia* [18].

Caesium trifluoroacetate density gradient ultracentrifugation was used to separate the ^13^C-labelled ‘heavier’ RNA from the un-labelled ^12^C ‘lighter’ RNA within the ^13^C-fed samples (Additional file 1: Fig. S1). RNA samples from the control ^12^C dietary treatment also underwent density gradient ultracentrifugation. The resulting gradients were fractionated by buoyant density ultracentrifugation, and cDNA from each fraction was used in quantitative RT-PCR reactions (Additional file 1: Figs. S3 & S4). This demonstrated that 16S rRNA gene transcripts had shifted to higher buoyant densities under the ^13^C dietary treatment (Additional file 1: Fig. S3). A peak in transcripts was detected at an average buoyant density of 1.789 g ml^−1^ (± 0.003 SD) under the ^12^C treatment, but this had shifted to a significantly higher buoyant density (1.797 g ml^−1^ ± 0.001, *P* = 0.016 in a two-sample *t*-test) under the ^13^C dietary treatment (Additional file 1: Fig. S4). This is consistent with the heavier ^13^C isotope being incorporated into the RNA of cuticular bacteria, due to the metabolism of labelled host resources (Additional file 1: Fig. S4).

**Fig. 2:**
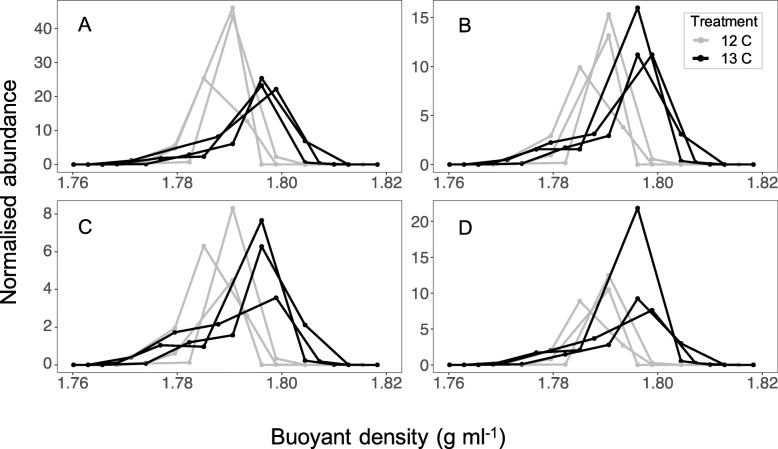
Normalised abundances of the genera *Pseudonocardia* (**A**), *Streptomyces* (**B**), *Microbacterium* (**C**), and *Wolbachia* (**D**) in buoyant density fractions of RNA samples taken from ants fed on a ^13^C (black) or ^12^C (grey) glucose diet. There were three replicate samples per treatment. The relative abundances of genera were normalised by multiplying by the percentage of 16S rRNA gene transcripts occurring in each fraction within a sample (see Additional file 1: Fig. S4)

Fractions spanning the peaks in transcript number were selected for 16S rRNA gene amplicon sequencing (Additional file 1: Fig. S4). The relative abundances of taxa identified by sequencing were further normalised using the percentage of 16S rRNA gene transcripts detected in each fraction in the qPCR experiments; this was to account for the differences in the absolute abundance of 16S rRNA gene transcripts detected in each fraction and also to facilitate the comparison of replicates within and across treatments, which differed in the total amount of RNA extracted. Sequencing confirmed that transcripts from actinobacterial genera had shifted to higher buoyant densities under the ^13^C treatment (Fig. 2A–C). For example, the abundance of the vertically transmitted *Pseudonocardia* symbiont tracked changes in the total number of 16S rRNA gene transcripts that had been identified using qPCR (Fig. 2A, Additional file 1: Fig. S4). Specifically, *Pseudonocardia* sequences were detected at an average relative abundance of 36.30% (± 7.22) in the peak fractions of the ^13^C treatment (average buoyant density of 1.797 g ml^−1^ ± 0.001, Fig. 2A)—these fractions contained an average of 66.45% (± 12.34) of the total number of 16S rRNA gene transcripts identified within a sample (Additional file 1: Fig. S4), giving a mean normalised abundance of 23.57 ± 1.67 (Fig. 2A). Although *Pseudonocardia* sequences were also detected in fractions of equivalent buoyant density under the ^12^C treatment (Fig. 2A), these fractions contained less than 2% of the total number of 16S rRNA gene transcripts within these samples (Additional file 1: Fig. S4); hence, a mean normalised abundance of 0.78 ± 1.29 was recorded (Fig. 2A). The abundance of *Pseudonocardia* transcripts instead peaked at a significantly lower buoyant density (1.789 g ml^−1^ ± 0.003, *P* = 0.016) under the ^12^C treatment.

In addition to *Pseudonocardia*, horizontally acquired taxa, including *Streptomyces* and *Microbacterium*, also showed similar shifts to higher buoyant densities under the ^13^C treatment and were abundant in peak fractions of ^13^C samples (Fig. 2B, C). For example, *Streptomyces* had an average normalised abundance of 12.790 ± 2.758 in peak fractions of the ^13^C samples. This indicates that ant-derived resources were not solely available to the *Pseudonocardia*, which would otherwise have dominated the ^13^C heavy fractions, but are available to, and taken up by, all bacteria on the cuticle.

The frequency of *Wolbachia* also shifted to heavier fractions under the ^13^C heavy treatment (Fig. 2D). Since *Wolbachia* are extracellular muscular tissue symbionts in this particular symbiosis [45], this finding supports the interpretation that resources were supplied to cuticular bacteria by the ant hosts and not taken directly from the glucose water. This interpretation is also backed by isotope ratio mass spectrometry (IRMS), which showed that surface-washed ants incorporate a significant amount of the ^13^C from their glucose diet into their bodies (Additional file 1: Fig. S5), and by direct fluorescent microscopy demonstrating that the glucose water was not transferred to the propleural plate (Additional file 1: Fig. S2). Note that abundances of all other taxa become ca. 20% and ca. 50% higher after excluding *Wolbachia* in the ^12^C and ^13^C treatment, respectively (Fig. 2), i.e. when considering only the cuticular microbiome.

### Antibacterial BGCs are expressed by *Pseudonocardia* on the ant cuticle

We previously generated high-quality genome sequences for five *Pseudonocardia octospinosus* and five *P. echinatior* strains isolated from *Acromyrmex echinatior* ant colonies and identified several BGCs (biosynthetic gene clusters) in each of their genomes that are associated with antimicrobial activity [32]. To establish if these BGCs are expressed in vivo on the ant cuticle, total RNA was extracted and sequenced from the propleural plates of ants in the captive colonies Ae088 (which hosts a vertically transmitted *P. echinatior* strain) and Ae1083 (which hosts a *P. octospinosus* strain) (Additional file 1: Table S1 [32, 37, 47–56]). A single RNA extraction was carried out for each colony, with each sample consisting of the pooled propleural plates of 80 individual ants. RNA samples were sequenced, and the resulting reads were quality filtered and mapped to their corresponding *Pseudonocardia* reference genomes [32] (Additional file 1: Table S2). Both *Pseudonocardia* species showed very similar patterns of gene expression in vivo, with genes involved in the production of secondary metabolites, including antibiotics (as classified by KEGG) being expressed at similar levels by both *Pseudonocardia* strains on the cuticle of *A. echinatior* ants (Additional file 1: Fig. S6).

**Fig. 3:**
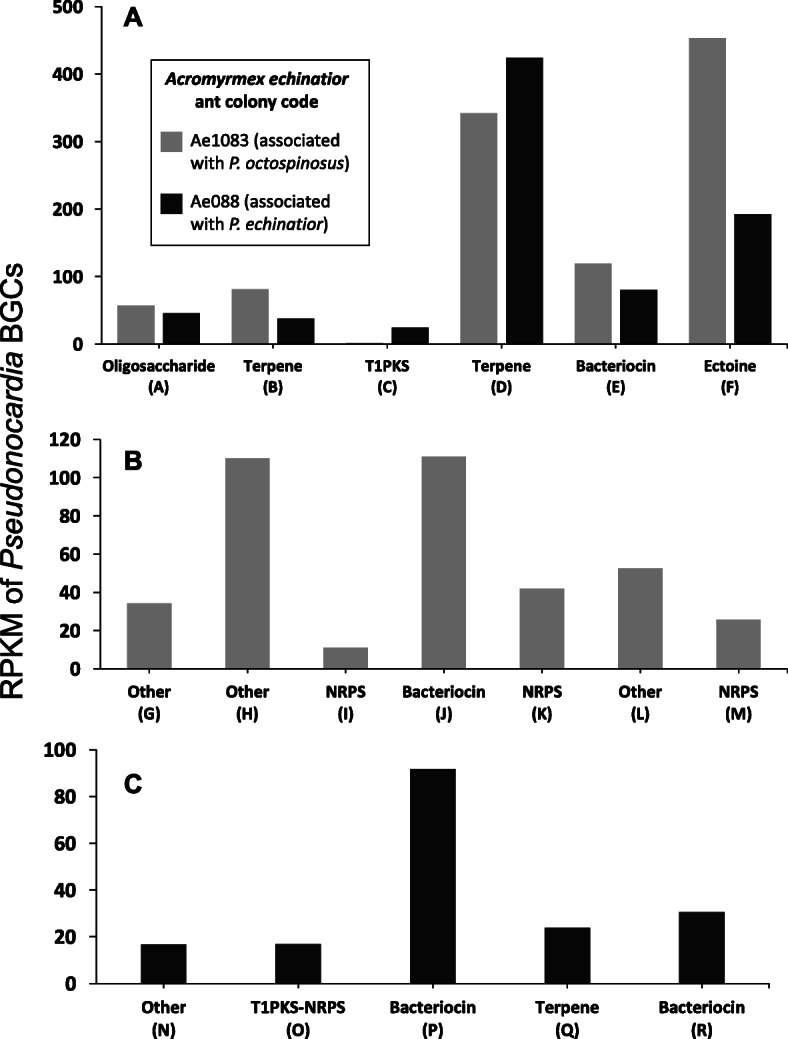
Expression (in reads per kilobase of transcript per million mapped reads (RPKM)) of **A** biosynthetic gene clusters that are shared between the two *Pseudonocardia* species, **B** biosynthetic gene clusters that are unique to *P. octospinosus* (colony Ae1083), and **C** biosynthetic gene clusters that are unique to *P. echinatior* (colony Ae088). Biosynthetic gene cluster codes relate to Additional File 1: Table S3. The results from each colony were derived from a pool of 80 ants

BGCs that are shared by the *P. octospinosus* and *P. echinatior* strains (Additional file 1: Table S3 [32]) displayed remarkably similar patterns of in situ expression on the propleural plates (Fig. 3A). For both *Pseudonocardia* species, the most highly expressed BGCs encoded proteins responsible for the synthesis of the compound ectoine and a putative carotenoid terpene pigment (cluster D and F, Fig. 3A). Such compounds are known to provide protection against abiotic stressors such as desiccation and high concentrations of free radicals which are often associated with biofilms [57–59]. Also expressed on the propleural plates is a shared BGC encoding a putative bacteriocin (cluster E, Fig. 3A), which belongs to a family of ribosomally synthesised post-translationally modified peptide (RiPP) antibiotics produced by many species of bacteria [60–62]; these are known to prevent the formation of biofilms by other microbial species [60, 63]. In contrast, a shared type 1 PKS gene cluster, encoding nystatin-like antifungal compounds [32, 37], had very low expression in the *P. echinatior* strain and was not expressed at all in the *P. octospinosus* strain (cluster C, Fig. 3A). This suggests that additional cues, such as direct exposure to *E. weberi* may be required to activate this BGC, since both *Pseudonocardia* strains produce inhibitory antifungals when confronted with *E. weberi* in vitro (Additional file 1: Fig. S7). A *Pseudonocardia* strain isolated from *Acromyrmex* ants has also previously been shown to produce nystatin-like compounds in vitro [37]. The most highly expressed BGCs unique to *P. echinatior* (cluster P, Fig. 3C) or *P. octospinosus* (cluster J, Fig. 3B) are both predicted to encode bacteriocins.

**Fig. 4:**
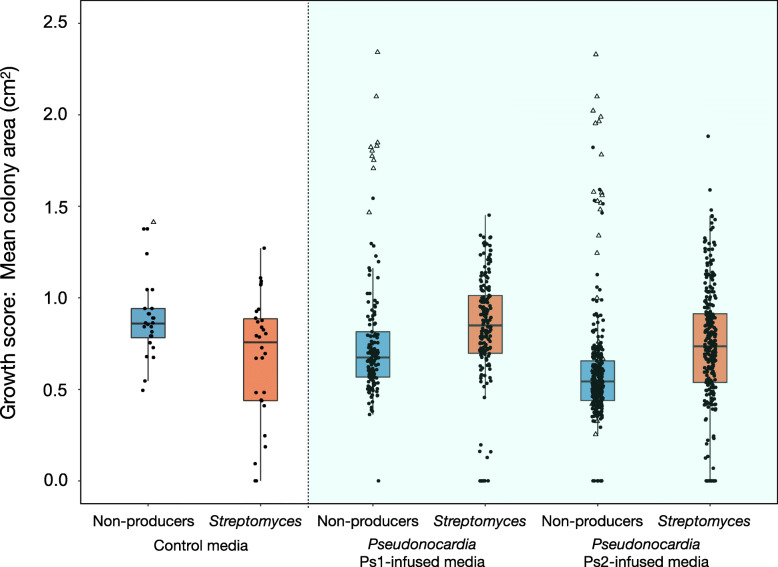
Growth rate experiments. Bacterial colony sizes after 5 days at 30 °C. Boxplots indicate medians ± one quartile. The white section shows the growth rates on control media, and the coloured section shows the growth rates on the *Pseudonocardia*-infused media. Red boxes represent non-antibiotic-producer strains, and blue boxes represent antibiotic-producing *Streptomyces* strains. A linear mixed-effects model [100], including 17 *Pseudonocardia* strains and 20 inoculated bacterial species as random intercepts, was used to test for interaction and main effects of growth media (control vs. Ps1-infused vs. Ps2-infused) and antibiotic production (non-producers vs. producers): *lme4::lmer*(Growth score ~ Growth media × Antibiotic production + (1|Ps strain/Plate) + (1|In strain)). One non-producer strain (*Staphylococcus epidermidis*) grew more rapidly than all other strains (open triangle points), demonstrating the need to control for correlated residuals

Taken together, the results of the RNA-SIP and RNA sequencing experiments are consistent with both previous empirical research [18, 33] and the screening hypothesis [12, 13]: the ant host provides public resources to its cuticular microbiome via glandular secretions [41] for which colonising ectosymbionts may compete. This is always after the native *Pseudonocardia* has established and gained dominance, which creates a demanding cuticular environment for any additional strain to invade.

### *Pseudonocardia* antibacterials create a demanding environment for non-antibiotic-producing bacteria

Next, we compared the growth rates of antibiotic-producing *Streptomyces* strains and non-antibiotic-producing bacteria on antibiotic-infused and control media. All strains were isolated from soil or fungus-growing ant nests (Additional file 1: Table S1). The antibiotic-infused media were created by growing lawns of 17 *Pseudonocardia* isolates (Additional file 1: Table S1) on SFM agar, while control media were inoculated with 20% glycerol. After a 6-week incubation period, the agar medium was flipped to reveal a surface for colonisation. The non-producer strains grew more quickly on the non-demanding control media while the antibiotic-producers grew more quickly on the demanding *Pseudonocardia*-infused media, producing a highly significant statistical interaction effect (*n* = 975, *χ*^2^ = 45.86, df = 2, *P* < 0.0001; Fig. 4). There was also a significant main effect of *Pseudonocardia* genotype, with both non-producers and producers exhibiting a lower growth rate on *P. echinatior* (Ps2)-infused media than on *P. octospinosus* (Ps1)-infused media (linear mixed-effects model, *n* = 915, *χ*^2^ = 24.55, df = 1, *P* < 0.0001, control-media data omitted for this analysis). This outcome is consistent with the observation by Andersen et al. [18] that *Acromyrmex* colonies hosting Ps2-dominated cuticular microbiomes were less prone to secondary invasion by other bacteria.

**Fig. 5:**
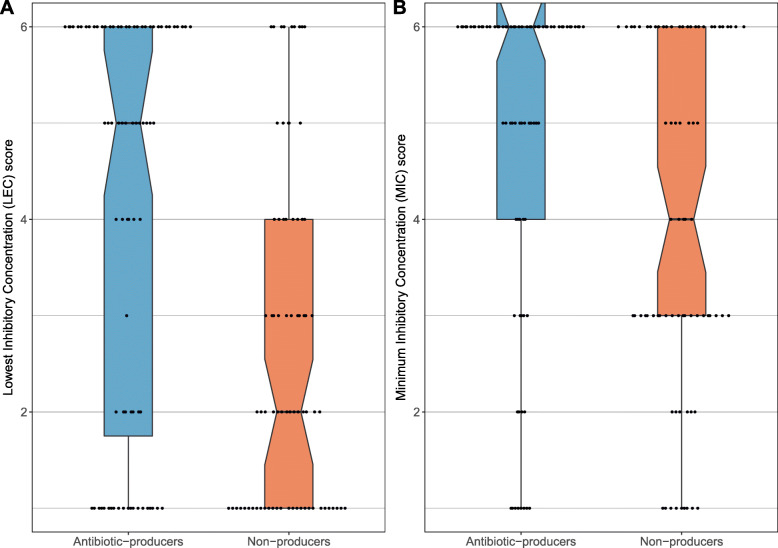
Antibiotic resistance profiles (**A** LEC and **B** MIC) for producer and non-producer strains (Additional file 1: Table S1), based upon each strain’s mean growth score across the eight tested antibiotics (details in Additional file 1: Table S4). Boxplots indicate the medians (notches) ± one quartile. ‘Rabbit ears’ in **B** indicate that the medians are also the highest values

To test the hypothesis that producer strains are generally resistant to antibiotics, which would confer competitive superiority in an antibiotic-infused host environment [12], we grew ten producer strains (all *Streptomyces* spp.) and ten non-producer strains (Additional file 1: Table S1) in the presence of eight different antibiotics (Additional file 1: Table S4), representing a range of chemical classes and modes of action. After 7 days, the lowest effective concentration (LEC, lowest concentration with inhibitory effect) and minimum inhibitory concentration (MIC, lowest concentration with no growth) scores were assigned on a Likert scale of 1–6, where a score of 1 was no resistance and a score of 6 was resistance above the concentrations tested [64]. Antibiotic-producer strains exhibited greater levels of resistance, measured by both LEC (Wilcoxon two-sided test, *W* = 94.5, *P* = 0.0017) and MIC scores (*W* = 80, *P* = 0.0253); *P*-values were corrected for two tests (Fig. 5).

We also performed growth rate experiments and measured antibiotic resistance profiles with resident non-antibiotic-producing strains that had been directly isolated from cuticular microbiomes. These strains had significantly slower growth rates overall, even on control media without antibiotics, suggesting that these strains are just transient environmental contaminants (Additional file 1: Fig. S8). These resident non-producer strains also demonstrated high levels of resistance as expected (Additional file 1: Fig. S9), given that they had been isolated from ant cuticles.

### *Pseudonocardia* antibacterials allow *Streptomyces* to competitively exclude non-antibiotic-producing bacteria

**Fig. 6:**
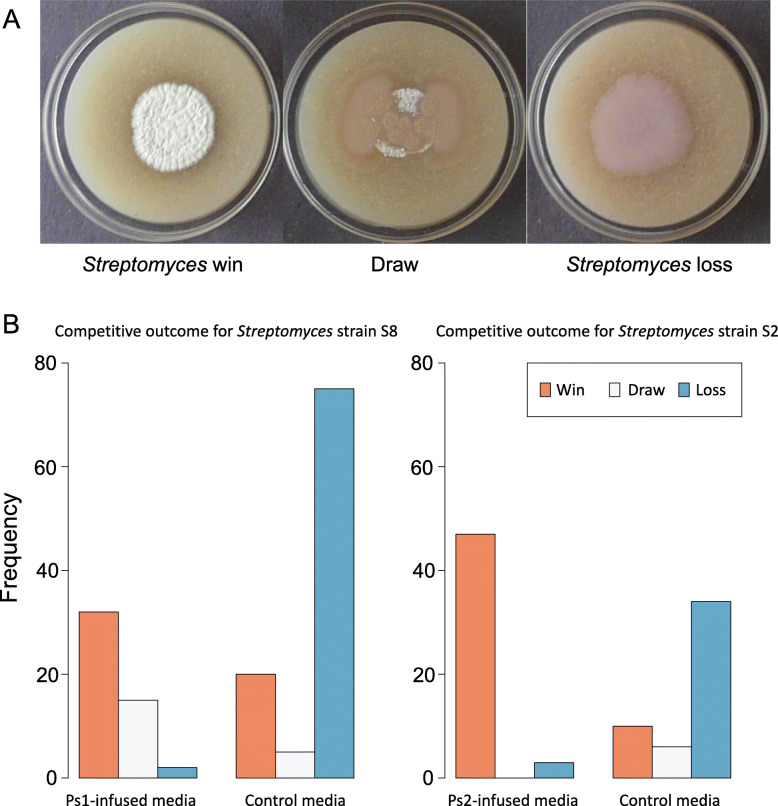
Pairwise competition experiment, scoring the frequency of producer wins. **A** Representative images of agar plates at 5 days post-inoculation showing examples of the three competitive outcomes: win (producer S2 vs. non-producer St3 on Ps2 media), loss (producer S8 vs. non-producer St3 on control media), and draw (producer S2 vs. non-producer St3 on control media) (strain details in Additional file 1: Table S1). **B** Bar charts of competitive outcomes for the two *Streptomyces* producer strains (S8 and S2; Additional file 1: Table S1). Each *Streptomyces* strain was individually competed against ten different non-antibiotic-producer strains. *Streptomyces* is more likely to win on *Pseudonocardia*-infused media. A general linear mixed-effects model, with ten non-antibiotic-producer strains as a random intercept, was used to test for the effect of medium (control vs. Ps-infused) on the competitive outcome (win vs. loss). For analysis, draws were omitted. S8 on Ps1-infused media: *n*_Ps1-infused_ = 34, *n*_Control_ = 95, *χ*^2^ = 103.6, df = 1, *P* < 0.0001; S2 on Ps2-infused media: general linear mixed-effects model, *n*_Ps2-infused_ = 50, *n*_Control_ = 44, *χ*^2^ = 87.9, df = 1, *P* < 0.0001. *lme4::glmer*(outcome ~ medium + (1 | non-producer strain), family = binomial)

Finally, to test whether producer strains have a competitive advantage in the demanding environment created by *Pseudonocardia*, we pairwise-competed two of the *Streptomyces* producer strains, named S2 and S8 (Additional file 1: Table S1), against each of 10 environmental non-producer strains on normal and on antibiotic-infused media. In the latter case, *Pseudonocardia* was again grown on agar plates before turning the agar over and coinoculating producer and non-producer test strains. On normal growth media, S*treptomyces* were more likely to lose to non-producers, but on *Pseudonocardia*-infused media, *Streptomyces* were more likely to win (general linear mixed-effects model; S8 on Ps1-infused media: *n* = 129, *χ*^2^ = 103.6, df = 1, *P* < 0.0001; S2 on Ps2-infused media: general linear mixed-effects model, *n* = 94, *χ*^2^ = 87.9, df = 1, *P* < 0.0001; Fig. 6).

## Discussion

We tested screening theory using the external (cuticular) microbiome of the leaf-cutting ant *Acromyrmex echinatior* as an experimental model. We used RNA-SIP to show that an animal host is directly feeding its microbiome (Fig. 2). We further show that the resource is public, meaning that the resource is used for growth not only by vertically transmitted *Pseudonocardia* but also by multiple species of environmentally acquired bacteria on the ant cuticle (Fig. 2). We then demonstrated, in two separate ant colonies, that both *P. octospinosus* and *P. echinatior* strains express antibacterial biosynthetic gene clusters (BGCs) on the ant cuticle (Fig. 3). We next showed that the two species of actinobacteria have broad-spectrum antibacterial activity against environmental isolates in vitro and, importantly, have a weaker effect on *Streptomyces* than on non-producers (Fig. 4), consistent with these *Streptomyces* species being resistant to a range of antibiotics (Fig. 5), which is typical for this genus. Finally, we used in vitro competition experiments to demonstrate that *Streptomyces* species can competitively exclude faster-growing bacteria that do not make antibiotics, but only when the competing species are grown on media infused with *Pseudonocardia* metabolites (Fig. 6).

Although feeding treatments for the RNA-SIP experiment were conducted over a short time-frame (10 days), it is possible that some of the bacterial symbionts acquired resources indirectly, by using labelled metabolites produced by other microbes that were feeding directly on the ant-derived resources. However, cross-feeding is still consistent with the food resource being public, and thus consistent with competition-based screening, since multiple bacterial species, including horizontally acquired actinobacteria, are still taking up host-supplied resources for growth, albeit indirectly, thus fuelling competition amongst species for host space.

Taken together, these results are consistent with the hypothesis that competition-based screening is a plausible mechanism for maintaining the integrity of the co-adapted mutualism between the leaf-cutting ant farming symbiosis and its defensive microbiome, predicted to be conditional on the vertically transmitted *Pseudonocardia* symbiont always being the first to establish and create a demanding environment. This priority establishment is invariably the case because callow large workers are inoculated by their nestmates within 24 h of emerging from their pupae [34]. *Pseudonocardia* is also never competitively excluded from the microbiome, as predicted by Scheuring and Yu [12] and empirically shown by Andersen et al. [18]. Indeed, initial advantages of early colonisation and nutrients allow *Pseudonocardia* to form a dense growth before additional microorganisms can colonise the ant cuticle. The new results reported here illustrate the tractability of the cuticular leaf-cutting ant microbiome, which is accessible to experimentation and for which the adaptive benefit to the ant hosts is clearly defined and explicitly testable: defence against specialised *Escovopsis* pathogens and colony collapse [3, 25, 37, 65]. Our present results are consistent with our previous hypothesis that the vertical transmission of *Pseudonocardia* results in *Streptomyces* strains being superior contenders for secondary acquisition [12].

An alternative hypothesis to screening is that the ants selectively acquire additional antibiotic-producing bacteria via a lock-and-key mechanism, in the same way that leguminous plants recognise *Rhizobium* symbionts via species-specific Nod-factor signalling molecules [66]. However, lock-and-key signalling requires tight coevolution between all candidate symbiont and host lineages (see [67] for one model), which could be true for the *Acromyrmex*- associated *Pseudonocardia* species but is highly unlikely for the other genera across the phylum Actinobacteria that can become established on the ant cuticle (Fig. 1). In contrast, legumes associate with only one genus of root-nodule symbionts, *Rhizobium*.

The petri dish competition experiments have the important advantage of allowing unambiguous scoring of wins, losses, and draws on media with and without *Pseudonocardia*. However, future work should focus on adding realism, since on the cuticle, competition is taking place amongst multiple species, with variation in colonisation order, the cuticular microenvironment, and resource provisioning rates [13]. However, although it is possible to prevent the establishment of *Pseudonocardia* on newly eclosed workers [34], the technical challenge will be to score relative species abundances (i.e. winners and losers) from sequencing datasets, in the face of cryptic species biases (see [68] for a discussion of this problem and [69] for a potential solution). A complementary approach would be to compare competition in vitro and in vivo with wild-type and knock-out strains of *Pseudonocardia* that are unable to produce the antimicrobial compounds observed in RNA-seq experiments. However, such an experiment would require extensive genetic modification, which has so far proven very challenging in *Pseudonocardia*.

Although competition has not been demonstrated via addition or removal experiments, we have shown clear resource-use overlap via the RNA-SIP experiment (Fig. 1) and the capacity for competitive exclusion via the petri dish experiment (Fig. 6). Combined with the capacities for exponential growth and for antibiotic production in the *Streptomyces* strains, we have a strong expectation of both scramble and interference competition in the attine cuticular microbiome.

The nature and origin of the host-derived resources remain elusive. Previous studies have observed that *Pseudonocardia* grows in or above specialised cavities on the ant cuticle, called foveae, that are underlain by structures that appear to be exocrine glands [41]. However, it has not been experimentally confirmed that these glands are the source of resources supplied to the cuticular microbiome. Another study has shown that metapleural gland secretions (the only other exocrine secretion that could reasonably spread over the entire cuticle) have no influence on the early exponential growth phase of *Pseudonocardia* in callow ants [70]. Closure of these glands was shown to influence the growth of *Pseudonocardia* in older ants, but it was unclear if artificial manipulations had compromised other aspects of ant health in this instance [70].

There are several emerging techniques that might be used to locate and identify host-derived substrates in future experiments. For example, high-resolution secondary ion mass spectrometry imaging (NanoSIMS), combined with fluorescent in situ hybridisation (FISH) can be used to directly visualise the assimilation of stable isotopes by different bacterial taxa [71]. This technique has been used to visually track the bacterial metabolism of labelled compounds secreted by the mouse intestinal mucosa [72]. Similarly, Raman microspectroscopy creates a chemical fingerprint of a molecule or system and can identify compounds that have incorporated heavy isotopes via the spectral shifts that take place [73]. Techniques such as matrix-assisted laser desorption/ionisation time-of-flight (MALDI-TOF) imaging mass spectrometry could also be applied in a similar way and, although extremely challenging to carry out, has been used to image the distribution of an antifungal compound over the surface of *A. echinati*or ants [39].

Our results show that competition-based screening is a plausible mechanism for the acquisition of a diverse, antibiotic-producing microbiome. The natural follow-on question is whether this mechanism represents an *Acromyrmex* adaptation that improves defense against pathogens. We do know that *Streptomyces* species isolated from *Acromyrmex* ants can produce antifungals that inhibit the growth of *Escovopsis* in vitro [37–39, 47], but a direct test would require experimental removal and addition of *Streptomyces* (and/or antibiotic gene knock-outs), which so far have posed significant technical challenges. However, as noted above, *Streptomyces* symbionts have rarely been found on the callow workers [18] that tend the fungus garden [35]. Thus, the screening effect shown in our experiments might be an epiphenomenon of the mutualism between *Pseudonocardia* and *Acromyrmex*. In this case, the leafcutter ant system can be thought of as a tractable model for a screening phenomenon that may have adaptive significance elsewhere. Alternatively, the adaptive benefit of the extra actinobacterial species could be that they improve the protection of foraging workers from bacterial and fungal infections acquired outside the colony, thereby also indirectly protecting workers that interact more extensively with the fungus garden. Given that bacterial competition is known to stimulate antimicrobial export in vitro [74, 75], having multiple strains on individual ants may then be beneficial by stimulating the production of multiple different antimicrobial compounds. Indeed, Schoenian et al. [39] have directly visualised the production of the antimicrobial valinomycin by a *Streptomyces* strain on the cuticles of mature *Acromyrmex* workers. We also hypothesise that continuous competition against antibiotic-producing competitors may select against *Pseudonocardia* losing costly antimicrobial production genes as a side effect of its domestication by attine ants.

Our results are of broad significance because competition-based screening provides a mechanistic explanation for microbiomes to be evolutionary stable ecosystems-on-a-leash [8]; here, the host leash works by fomenting and biassing competition, via the combination of public resources and a vertically transmitted antibiotic-producing symbiont, to favour the establishment of antibiotic-producing bacteria [12]. This perspective is consistent with the idea that host-associated microbiomes can have both core members that co-adapt with host environments and non-adapted but still mutualistic members. Studies in other symbioses appear to support this dual evolutionary and ecological view [14, 16, 17], both for an array of mutualistic symbioses with multicellular partners and for microbiomes more specifically. For instance, the actinomycete *Bifidobacterium longum* subsp. *infantis* dominates the guts of human neonates [11, 76]. In this case, the *Bifidobacterium* is an early coloniser because it is transmitted vertically from mother to child, and *Bifidobacterium* consumes a range of oligosaccharides provided in human breast milk to build up a large enough population that it can competitively exclude pathogen colonisers. We hypothesise that *Bifidobacterium* could act like *Pseudonocardia* and selectively favour the establishment of other *Bifidobacterium* species in the gut, at least until weaning. Similarities may also be seen in the ant species, *Lasius fuliginosus*, which stabilises its carton nest structures through the growth of a remarkably predictable community of fungi [77]. Experiments have shown that antimicrobial substances, originating from the ant body parts, are tolerated by these fungal associates and support their growth, enabling them to outcompete other species of doubtful loyalty to the symbiosis in the nest structure [77]. Similar processes may also play out during the establishment of plant root microbiomes [78–81] and could be open to manipulation in efforts to improve crop yields [82].

Future work on the *Acromyrmex* model system should include characterising the antibacterial molecules made by the symbiotic *Pseudonocardia* strains in vitro and in vivo and matching these compounds to the BGCs expressed on the ants. This will be challenging because ant-derived *Pseudonocardia* strains grow poorly on agar plates and rarely in liquid culture. Additionally, imaging mass spectrometry has, as yet, not been possible on the ants themselves.

## Methods

### Ant colony collection and maintenance

Colonies of *A. echinatior* (Hymenoptera, Formicidae, Attini) were collected from the Gamboa area of the Soberania National Park, Panama, between 2001 and 2014. Colonies Ae1083 and Ae088 (Additional file 1: Table S1) were maintained under controlled temperature conditions (25 °C) at UEA and fed a daily diet of bramble and laurel leaves. Additional colonies were maintained at the University of Copenhagen in rearing rooms at ca 25 °C and 70% relative humidity, where they were fed with bramble leaves and occasional supplements of apple and dry rice.

### RNA-stable isotope probing

#### RNA SIP ^13^C feeding experiment

Six replicate groups of 22 mature worker ants with visible bacterial growth on their propleural plates were selected from colony Ae1083 (Additional file 1: Table S1) and placed into 9-cm petri dishes containing a 2 × 2 cm square of cotton wool soaked in water. Following 24 h of starvation, three replicate groups of 22 ants were supplied with 300 μl of a 20% ^13^C glucose solution (w/v, Sigma Aldrich), and the remaining three groups were supplied with 300 μl of a 20% ^12^C glucose solution (w/v, Sigma Aldrich) for 10 days. Glucose solutions were supplied to ants in microcentrifuge tube caps and were refreshed every 3 days. To confirm the uptake of the ^13^C isotope by ants fed on the ^13^C labelled diet, a further 5 ants were fed on each type of glucose diet; these were submitted for isotope ratio mass spectrometry (IRMS) analysis, which enables the relative abundance of each stable isotope (^13^C and ^12^C) to be quantified in a sample.

#### Isotope ratio mass spectrometry analysis

The ^13^C composition of ants fed on a ^13^C-labelled diet was determined by using a coupled Delta plus XP Isotope Ratio Mass Spectrometer/Flash HT Plus Elemental Analyser (Thermo Finnigan) in the University of East Anglia Analytical Facility. Ants were fed on a 5% glucose solution (w/v) for 10 days; five ants were fed a ^13^C glucose solution, and five were fed on a ^12^C glucose solution. After 10 days, ants were washed once in 70% ETOH, then sequentially in sterile dH_2_O before drying on filter paper. The ants were then flash frozen and stored at − 80 °C until being placed in a ScanVac Coolsafe freeze dryer for 5 days. Each ant was then put into an individual 75-μl tin capsule (Elemental Microanalysis); capsules were loaded into an automatic sampler and completely converted to CO_2_, N_2_ and H_2_O through combustion in an excess of oxygen (oxidation was carried out at 1020 °C, followed by reduction at 650 °C). Nitrous oxides formed during combustion were reduced using Cu. Helium was used as a carrier gas. After passing through a water trap (MgClO_4_), the gases were separated chromatographically on an isothermal GC column (Thermo PTFE, 0.8 m, 50 °C); the resulting peaks sequentially entered the ion source of the isotope ratio mass spectrometer. Gas species were then measured using a Faraday cup universal collector array, with masses of 44, 45, and 46 being monitored for the analysis of CO_2_. Casein and collagen were used to calibrate the system and normalise the data post-run; these standards have been calibrated against international certified standards and have an assigned δ^13^C value. Empty tin capsules were used as blanks. Each sample was analysed in triplicate. The ^13^C content of samples was reported as the ^13^C atom percent, which was calculated using the following formula: (^13^C/^12^C + ^13^C) × 100

#### Fluorescent microscopy of ant feeding habits

To confirm that the glucose water diet did not spread over the ant cuticle, a 20% glucose solution labelled with non-toxic fluorescent green drain tracing dye (Hydra) was fed to ants. Five mg ml^−1^ of dye was added to a 20% glucose solution, of which 300 μl was supplied to ants in the cap of an Eppendorf. Ants were sampled just after taking a feed, and after 6 and 24 h of being exposed to the dye, to trace the spread of the solution over time. After sampling, ants were carefully fixed on their backs, and brightfield and fluorescent images were acquired using a Zeiss M2 Bio Quad SV11 stereomicroscope (Additional File 1: Fig. S2). The samples were illuminated either with a halogen lamp (brightfield) or a 100-W Hg arc lamp (fluorescence) and reflected-light images were captured with an AxioCam HRc CCD camera and AxioVision software (Carl Zeiss, Cambridge, UK). Green fluorescence was excited with light passed through a 470-nm filter (40 nm bandpass), and the emission was collected through a 525-nm filter (50 nm bandpass).

#### Cuticular dissection and RNA extraction

At the end of the 10-day feeding experiment, the propleural plates of the propleura were removed from the ventral exoskeleton using a dissection microscope and fine sterile tweezers. Propleural plates from each of the 22 ants in each dietary group were placed together in lysis matrix E tubes (MP Biomedicals) on dry ice, before being snap-frozen in liquid nitrogen. A modified version of the Qiagen RNeasy Micro Kit protocol was used for all RNA extractions. Briefly, 700 μl of RLT buffer (with 1% beta mercaptoethanol) was added to each lysis matrix E tube before the samples could thaw. Tubes were then placed in a FastPrep-24™ 5G benchtop homogeniser (MP Biomedicals) and disrupted for 40 s at 6 m/s. Samples were then centrifuged for 2 min at 13,000 rpm, and the supernatant was collected into a QIAshredder tube. This was centrifuged for 2 min at 13,000 rpm to homogenise the lysate. The resulting flow-through was mixed vigorously with 700 μl acidic phenol chloroform, then allowed to rest for 3 min at room temperature before centrifugation for 20 min at 13,000 rpm. The upper phase was then collected, and a 50% volume of 96% ethanol was added. The mixture was then placed into a MinElute column supplied with the Qiagen RNeasy Micro Kit. The kit protocol (including the on-column DNase I treatment) was then followed through to elution of the RNA, at which point 50 μl of RNase free water (heated to 37 °C) was added to the column membrane and incubated at 37 °C for 5 min, before centrifuging for 1 min at 13,000 rpm to elute the RNA. To remove any remaining DNA, RNA was treated with the turbo DNase kit (Invitrogen): 5 μl of 10× buffer and 2 μl of Turbo DNase were added to 50 μl of RNA and incubated at 37 °C for 25 min. RNA was then purified using the Qiagen Micro RNA Kit clean-up protocol. The quantity and purity of all RNA samples were checked using a nanodrop spectrophotometer and a Qubit™ RNA HS assay kit (Invitrogen™).

#### Density gradient ultracentrifugation and fractionation

Density gradient ultracentrifugation was carried out to separate ^13^C labelled (‘heavy’) from un-labelled (‘light’) RNA within the same sample. To make one complete gradient solution for the ultracentrifugation of one RNA sample, 4.5 ml of caesium trifluoroacetate (CsTFA, ~ 2 g ml^−1^, GE Healthcare, Munich, Germany) was added to 850 μl of gradient buffer and 197.5 μl formamide. Gradient buffer was made using an established protocol [83]. Following this, 270 ng of RNA from one replicate sample was added to the gradient solution, and the refractive index (R.I) of a 60 μl aliquot was measured using a refractometer (Reichert Analytical Instruments, NY, USA). The R.I was then normalised to 1.3725 (approximately 1.79 g ml^−1^ CsTFA). The samples underwent ultracentrifugation in a Beckman Optima XL-100K ultracentrifuge for 50 h at 20 °C, 38,000 rpm with a vacuum applied, using a Vti 65.2 rotar (Beckman Coulter, CA, USA). Deceleration occurred without brakes. Following centrifugation, samples were divided into 12 fractions using a peristaltic pump to gradually displace the gradient, according to an established protocol [44]. The R.I of fractions was measured to confirm the formation of a linear density gradient. RNA was precipitated from fractions by adding 1 volume of DEPC-treated sodium acetate (1 M, pH 5.2), 1 μl (20 μg) glycogen (from mussels, Sigma Aldrich), and 2 volumes of ice-cold 96% ethanol. Fractions were incubated overnight at − 20 °C then centrifuged for 30 min at 4 °C at 13,000×*g*, before washing with 150 μl of ice-cold 70% ethanol and centrifuging for a further 15 min. Pellets were then air-dried for 5 min and re-suspended in 15 μl of nuclease-free water.

#### Quantifying 16S rRNA gene copy number across RNA SIP fractions

The RNA in each fraction was converted to cDNA by following the manufacturer’s instructions for Superscript II (Invitrogen) with random hexamer primers (Invitrogen). 16S rRNA gene copy number was then quantified across cDNA fractions using qPCR. For this, 1 μl of either template cDNA, standard DNA, or dH_2_O as a control, was added to 24 μl of reaction mix containing 12.5 μl of 2× Sybr Green Jumpstart Taq Ready-mix (Sigma Aldrich), 0.125 μl of each of the primers PRM341F and 518R (Additional File 1: Table S1), 4 μl of 25 mM MgCl2, 0.25 μl of 20 μg μl^−1^ Bovine Serum Albumin (Sigma Aldrich), and 7 μl dH_2_O. Sample cDNA, standards (a dilution series of the target 16S rRNA gene at known quantities), and negative controls were quantified in duplicate. Reactions were run under the following conditions: 95 °C for 10 min; 40 cycles of 95 °C for 15 s, 55 °C for 30 s, and 72 °C for 30 s; plate read step at 83.5 °C for 10 s (to avoid primer dimers); 96 °C for 15 s; 100 cycles at 55–95 °C for 10 s, ramping 0.5 °C per cycle, followed by a plate read. Reactions were performed on 96-well plates (Bio-Rad). The threshold cycle (CT) for each sample was then converted to target molecule number by comparing to CT values of a dilution series of target DNA standards. These values were further converted to percentages based on the total number of 16S rRNA gene transcripts identified in each sample.

#### Sequencing and analysis

PCR was used to amplify the 16S rRNA gene in each of the fractions that spanned the peaks in 16S rRNA gene copy number, identified via qPCR. This was done using the primers PRK341F and 518R (Additional file 1: Table S1). One unfractionated sample was also created for ants under each of the ^13^C or ^12^C dietary treatments, by pooling equal quantities of unfractionated cDNA from each of the 3 replicate groups and using this as a template for PCR amplification. The resulting PCR products were purified using the Qiagen MinElute™ gel extraction kit and submitted for 16S rRNA gene amplicon sequencing using an Illumina MiSeq at MR DNA (Molecular Research LP), Shallowater, TX, USA. Sequence data was then processed by MR DNA using their established pipeline (as described in [84, 85]). As part of this pipeline, paired-end sequences were merged, barcodes were trimmed, and sequences of less than 150 bp and/or with ambiguous base calls were removed. The resulting sequences were denoised, and OTUs were assigned by clustering at 97% similarity. Chimeras were removed, and OTUs were taxonomically assigned using BLASTn against a curated database from GreenGenes, RDPII, and NCBI [86]. Plastid-like sequences were removed from the analysis. Upon receipt of the 16S rRNA gene sequencing data from MR DNA, OTU assignments were verified using QIIME2 and BLASTn, and statistical analysis was carried out using R 3.2.3[87]. OTUs assigned as *Pseudonocardia* were blasted against the 16S rRNA gene sequences for *P. echinatior* and *P. octospinosus* [32] to confirm the relative abundance of each of these vertically transmitted strains in the samples. All 16S rRNA gene amplicon sequencing data from this experiment has been deposited in the European Nucleotide Archive (ENA) public database under the study accession number PRJEB32900 [88]. Relative abundances were normalised using the qPCR data on the total 16S rRNA gene transcripts occurring within a fraction. Specifically, the following formula was used: (*R* × *P*)/100, where *R* is the relative abundance of a taxon and *P* is the percentage of 16S rRNA gene transcripts detected in that particular fraction of a sample.

### Detecting the expression of *Pseudonocardia* BGCs in situ

#### RNA extraction and sequencing from ant propleural plate samples

The propleural plates of *Acromyrmex echinatior* ants were dissected (as described above) from individual mature worker ants that had a visible growth of *Pseudonocardia* bacteria on their cuticle. A pool of 80 ant cuticles was sampled from each of the colonies Ae1083 and Ae088, respectively (Additional file 1: Table S1), after which RNA was extracted as described above. The quantity, purity, and integrity of all RNA samples were checked using a nanodrop spectrophotometer and Qubit™ RNA HS assay kit (Invitrogen™), as well an Experion™ bioanalyser with a prokaryotic RNA standard sensitivity analysis kit (Bio-Rad, California, USA). One microgram of RNA from each of the propleural plate samples was sent to Vertis Biotechnologie AG (Freising-Weihenstephan, Germany) where samples were processed and sequenced using an RNA sequencing approach [as described in [89]. Single-end sequencing (75 bp) was performed using an Illumina NextSeq500 platform. All sequencing reads have been deposited in the ENA public database under the study accession number PRJEB32903 [90].

#### Processing of reads generated from RNA sequencing experiments

The quality of Illumina sequences (returned from Vertis Biotechnologie AG) was assessed using the program FastQC (Babraham Institute, Cambridge, UK), before using TrimGalore version 0.4.5 (Babraham Institute, Cambridge, UK) to trim Illumina adaptors and low-quality base calls from the 3′ end of reads (an average quality phred score of 20 was used as cut-off). After trimming, sequences shorter than 20 base pairs were discarded. Trimmed files were then aligned to the reference genome for *Acromyrmex echinatior* (Additional File 1: Table S1 [55]) and the appropriate *Pseudonocardia* genome (either Ae707 for the sample from colony Ae1083, or Ae706 for the sample from colony Ae088 [32]; see Additional File 1: Table S1 for genome information). All alignments were done using the splice-aware alignment program HiSat2 [91] with the default settings. For each cuticular sample, reads that had mapped successfully to their respective *Pseudonocardia* genomes (Additional File 1: Table S2) were then mapped back to the ant genome (and vice versa) to check that reads did not cross-map between the two genomes (i.e. that they were either uniquely ant or bacterial reads)—reads that did not cross-map were retained for downstream analysis. Following alignment, the program HTSeq [92] was used to count mapped reads per annotated coding sequence (CDS) using the General Feature Format (GFF) file containing the annotated gene coordinates for each reference genome. Reads that mapped to multiple locations within a genome were discarded at this point, and only uniquely mapped reads were used in the counting process. Read counts per CDS were then converted to reads per kilobase of exon model per million reads (RPKM) by extracting the gene lengths from the GFF file. Converting reads to RPKM values normalises counts for RNA length and for the differences in sequencing depth, which enables more accurate comparisons both within and between samples [93].

#### Expression analysis

In order to investigate the expression levels of different functional groups of genes, protein sequences of every annotated gene in each *Pseudonocardia* genome [32] (Additional file 1: Table S1) were extracted and uploaded to BlastKOALA [94]. Assigned *K* numbers were classified into five main KEGG pathway categories (and their associated sub-categories) using the KEGG Pathway Mapper tool. Each gene, with its associated *K* number and category assignments, was then matched to its RPKM value from the RNA sequencing dataset so that the expression levels of different KO categories could be established. To investigate the expression of biosynthetic gene clusters (BGCs) by *Pseudonocardia* on the ant cuticle, reference genome sequences for *P. octospinosus* and *P. echinatior* (Additional file 1: Table S1 [32]) were uploaded to antiSMASH version 4.0, which predicts the presence and genomic location of BGCs based on sequence homology to known clusters [95]. RPKM values were then generated for each predicted BGC, based on the length of the predicted cluster and read counts for genes situated within it.

#### Isolation of *Pseudonocardia* and *Escovopsis* bioassays

The *Pseudonocardia* strains PS1083 and PS088 (Additional file 1: Table S1) were isolated from the propleural plates of individual large *Acromyrmex echinatior* workers taken from colonies Ae1083 and Ae088 (colonies used in RNA-seq experiments, see Additional file 1: Table S1), respectively. Similarly, *Pseudonocardia* strains Ae322, Ae712, Ae280, Ae160, Ae703, Ae702, Ae707, Ae704, and Ae715 were isolated from large worker ants from colonies with the same labels maintained at the University of Copenhagen (these strains were only used for the growth rate experiments described in the section below). A sterile needle was used to scrape bacterial material off the propleural plates on the ventral part of the thorax; this was then streaked over Soya Flour Mannitol (SFM, Additional File 1: Table S4) agar plates and incubated at 30 °C. The resulting colonies resembling *Pseudonocardia* were purified by repeatedly streaking single colonies onto SFM agar plates. Spore stocks were created using an established protocol [96]. The taxonomic identity of each *Pseudonocardia* isolate was confirmed via colony PCR and 16S rRNA sequencing, as described in Holmes et al. [32]. Each resulting sequence was also aligned to both the *Pseudonocardia octospinosus* and *Pseudonocardia echinatior* 16S rRNA gene sequences [32] to reveal their percentage identities to each of the two species.

Antifungal bioassays were carried out using the specialised fungal pathogen *Escovopsis weberi* strain CBS 810.71, acquired from the Westerdijk Fungal Biodiversity Institute (Additional File 1: Table S1). *E. weberi* was actively maintained on potato glucose agar (PGA, Additional File 1: Table S4) at room temperature. Fungal mycelia were transferred to a fresh plate every month. For bioassays, a plug of actively growing mycelium was transferred from PGA plates to the edge of a Glucose Yeast Malt (GYM, Additional File 1: Table S4) agar plate with a growing *Pseudonocardia* colony (strain PS1083 or PS088, Additional File 1: Table S1), using the end of a sterile glass Pasteur pipette. Plates were then left at room temperature for 2 weeks. A zone of clearing around the *Pseudonocardia* colony indicated the presence of antifungal activity. Three replicate experiments (with three replicate bioassay plates per *Pseudonocardia* strain) were carried out, whereby different *E. weberi* starter plates were used as an inoculum.

### In vitro competition experiments

#### Collection and isolation of bacterial strains

Nineteen strains of *Pseudonocardia* (11 strains of *P. echinatior* and 8 of *Pseudonocardia octospinosus*, Additional file 1: Table S1) were isolated from the cuticles of individual *Acromyrmex echinatior* worker ants across 18 different colonies and genotypes, as described above. Ten of these isolated *Pseudonocardia* strains were previously genome-sequenced by Holmes et al. [32]. The 10 environmental antibiotic-producer strains (all in the genus *Streptomyces*) were taken from general collections in the Hutchings lab and are a mixture of isolates from either soil environments or from worker ants taken from captive colonies (Additional file 1: Table S1). The 10 environmental non-producer strains were obtained from the Hutchings lab (two strains) and from the ESKAPE suite (eight strains with varying origins (human skin, soil, etc.)) used to test antibiotic resistance or efficacy in clinical/research settings (Additional file 1: Table S1).

#### Individual growth rate experiments

To create the *Pseudonocardia*-infused media, lawns of each of the 19 isolates (Additional File 1: Table S1) were grown, by plating 30 μl of spores (in 20% glycerol) onto 90 mm SFM agar plates (Additional File 1: Table S4). The control plates were inoculated with 20% glycerol only. We incubated these plates at 30 °C for 6 weeks, which ultimately produced confluent lawns from 17 strains that could be included in the experiments (6 Ps1, 11 Ps2). After a 6-week incubation period, the agar medium was flipped to reveal a new surface for colonisation. The 10 environmental producer strains and the 10 environmental non-producer strains (Additional File 1: Table S1) were then inoculated onto the plates to compare their growth rates on each type of media. Each of the plates received 10 evenly spaced colonies, with 3 replicates, generating 2 invader types × 17 Ps-media-types × 3 replicates = 102 Ps-infused plates, and 2 invader types × 3 replicates = 6 control plates, for 1020 treatment and 60 control inoculations. Each strain inoculation used 5 μl of solution (approx. 1 × 10^6^ cells per ml in 20% glycerol), spotted at evenly spaced positions and without coming into direct contact. All plates were incubated at 30 °C for 5 days, after which photographs were taken.

Images were processed in the Fiji software [97, 98], creating binary negatives (black and white) so automated tools could identify discrete areas of growth (black) and measure growth areas for each invading strain; in the few cases where binary image resolution was insufficient, outlines were added manually before area calculation. Forty-eight producer-inoculated and 57 non-producer-inoculated treatment measurements were excluded because plate condition had deteriorated to become unscorable or they were contaminated, leaving final sample sizes of 1020-48-57 = 915 treatment inoculations and 60 controls.

The second growth rate experiment compared the 10 *Acromyrmex*-resident, non-producer strains with 9 of the environmental producer strains (1 of the 10 inoculations failed to grow). All 19 *Pseudonocardia* strains grew sufficiently to be included in this experiment, and each plate was again inoculated with 10 or 9 evenly spaced colonies. Starting sample sizes were therefore 2 invader types × 19 Ps-media-types × 3 replicates = 114 Ps-infused plates and 2 × 3 = 6 control plates, for 1083 treatment and 57 control inoculations. Fifty producer and 20 non-producer treatment measurements were excluded for the same reasons as above, leaving final sample sizes of 1083-50-20 = 1013 treatment and 57 control inoculations, scored as above.

#### Pairwise competition experiment

Experiments were set up to test whether antibiotic-producer strains could win in direct competition against non-producing strains, both on normal media and on media infused with *Pseudonocardia* secondary metabolites. To create the *Pseudonocardia*-infused media, we plated 30 μl of spores (in 20% glycerol) onto 50 mm SFM agar plates (Additional File 1: Table S4). The control plates were inoculated with 20% (v/v) glycerol only. We incubated these plates at 30 °C for 6 weeks, which ultimately produced confluent lawns. As above, the agar was flipped to reveal a surface open for colonisation. Environmental producers and non-producers were then coinoculated onto these media (as well as on control media with no *Pseudonocardia* present), and we measured the outcome of competition as a win, loss, or draw. To keep the number of tests manageable, we used two combinations of *Pseudonocardia*-infused media and *Streptomyces*: *Pseudonocardia octospinosus* (strain Ae707-CP-A2) **+** *Streptomyces* S8, and *Pseudonocardia echinatior* (strain Ae717) **+** *Streptomyces* S2 (Additional File 1: Table S1). We competed the two *Streptomyces* strains (S2, S8) against the 10 environmental non-producer strains (20 pairings). Each *Strepromyces* strain was prepared as 10^6^ spores ml^−1^ in 20% glycerol. Each non-producer strains was grown overnight in 10 ml of Lennox broth (Additional File 1: Table S4), before subculturing (1:100 dilution) into 10 ml of fresh Lennox, and incubating at 37 °C for 3–4 h. The OD_600_ was then measured, assuming that an OD_600_ of 1 represented 8 × 10^9^ cells. Similar dilutions of 10^6^ cells per ml were made for each non-producer strain in 20% (v/v) glycerol, after which producer and non-producer preparations were mixed at a ratio of 1:1 (v/v) and co-inoculated as a mixture of 20 μl (10^4^ spore-cells of each) on the designated *Pseudonocardia*-infused media with 5 replicates per pairing. We used 150 plates for the S8 experiment (including 100 control plates; 10 replicates per pairing) and 100 plates for the S2 experiment (including 50 control plates; 5 replicates per pairing). Plates were incubated at 30 °C for 5 days before imaging, after which images were scored with respect to the producer as win (dominant growth), draw (both strains growing with no clear dominant), or lose (little or no visible growth), always with reference to images of each strain grown alone on control medium to minimise observer bias. One plate’s outcome was too ambiguous to score and was discarded. All plates were independently scored by two observers, one using photos of the original images, which produced datasets giving the same statistical results. We report the direct observer’s scores. For analysis, draw outcomes were omitted, and a general linear mixed-effects model, including non-antibiotic-producer strain as a random intercept (10 groups), was used to test for an effect of the medium term (control vs. Ps1/2-infused) on competitive outcome (win vs. loss) ((*lme4::glmer*(outcome ~ medium + (1 | non.producer.strain), family = binomial)). Significance was estimated using term deletion.

#### Antibiotic resistance assays

The key assumption of screening theory is that antibiotic-producers are better at resisting antibiotics, as measured by growth rates in the presence of antibiotics, because this correlation is what allows producer strains to better endure the demanding environment produced by *Pseudonocardia*. We tested this assumption by growing the 10 environmental producer strains, the 10 environmental non-producer strains, and the 10 resident non-producers strains (Additional File 1: Table S1) in the presence of 8 different antibiotics (Additional File 1: Table S4), representing a range of chemical classes and modes of action. Antibiotics were added to 1 ml of LB-Lennox/SFM medium (Additional File 1: Table S4) in a 24-well microtitre plate at 6 different concentrations. The relative concentration range was the same for each antibiotic, although actual concentrations reflected activity (Additional File 1: Table S4). Producers and non-producers were inoculated onto plates and incubated at 30 °C for 7 days, then photographed. The lowest effective concentration (LEC, lowest concentration with inhibitory effect) and minimum inhibitory concentration (MIC, lowest concentration with no growth) scores were assigned on a Likert scale of 1–6, where 1 was no resistance and 6 was resistance above the concentrations tested (adapted from generalised MIC methods; reviewed by Balouiri et al. [64]).

#### Statistical analyses

R markdown-format scripts, input datafiles, and html output files for the analyses in the main text Figures 4, 5, and 6 and in Figures S9 and S10 (Additional File 1) are provided as a single R project folder at github.com/dougwyu/Worsley_et_al_screening_test_R_code [99]

## Supplementary Information

**Additional file 1: **Supplemental tables, figures and methods referenced in the text. **Fig. S1.** Overview of methodology used for RNA stable isotope probing; **Fig. S2.** Fluorescent microscopy images of glucose water diet treatment; **Fig. S3.** The relationship between RNA-SIP fraction number and buoyant density; **Fig. S4.** 16S rRNA gene copy number across different fractions of buoyant density gradients, as determined via qPCR; **Fig. S5.** The atom percentage of ^13^C in ants, as determined by Isotope Ratio Mass Spectrometry (IRMS) analysis; **Fig. S6.** The expression of Kegg orthology pathway categories in *Pseudonocardia* symbiont strains; **Fig. S7.** The bioactivity of *Pseudonocardia* isolates against the specialized fungus-garden pathogen *Escovopsis weberi*; **Fig. S8**. Individual growth-rate experiments of *Acromyrmex*-resident, non-producer strains; **Fig. S9.** Antibiotic resistance profiles for producer, non-producer, and resident non-producer strains; **Table S1.** Details of ant colonies, bacterial and fungal strains, reference genomes and primers used in experiments; **Table S2.** Details RNA-sequencing reads from ant propleural plate samples; **Table S3.** Secondary metabolite BGCs in the *Pseudonocardia* mutualist genomes (table adapted from [32]); **Table S4**. Media recipes and antibiotics used in this study.

## Data Availability

The 16S rRNA gene amplicon sequencing data from the SIP experiment, as well as the RNA sequencing data, have been uploaded to the European Nucleotide Archive (ENA), under the accession numbers PRJEB32900 [88] and PRJEB32903 [90], respectively. R markdown-format scripts, input datafiles, and html output files for the analyses in the main text Figs. 4, 5, and 6 and in Additional file 1: Fig. S8 & S9 are also provided as a single R project folder at github.com/dougwyu/Worsley_et_al_screening_test_R_code [99].
