# Supplementary material for: Competition-based screening helps to secure the evolutionary stability of a defensive microbiome
Source: BMC Biol. 2021 Sep 15;19:205. doi: 10.1186/s12915-021-01142-w (PMC8444595; doi:10.1186/s12915-021-01142-w)
Supplement: Supplementary file 1 — Additional file 1: Supplemental tables, figures and methods referenced in the text. Fig. S1. Overview of methodology used for RNA stable isotope probing; Fig. S2. Fluorescent microscopy images of glucose water diet treatment; Fig. S3. The relationship between RNA-SIP fraction number and buoyant density; Fig. S4. 16S rRNA gene copy number across different fractions of buoyant density gradients, as determined via qPCR; Fig. S5. The atom percentage of 13C in ants, as determined by Isotope Ratio Mass Spectrometry (IRMS) analysis; Fig. S6. The expression of Kegg orthology pathway categories in Pseudonocardia symbiont strains; Fig. S7. The bioactivity of Pseudonocardia isolates against the specialized fungus-garden pathogen Escovopsis weberi; Fig. S8. Individual growth-rate experiments of Acromyrmex-resident, non-producer strains; Fig. S9. Antibiotic resistance profiles for producer, non-producer, and resident non-producer strains; Table S1. Details of ant colonies, bacterial and fungal strains, reference genomes and primers used in experiments; Table S2. Details RNA-sequencing reads from ant propleural plate samples; Table S3. Secondary metabolite BGCs in the Pseudonocardia mutualist genomes (table adapted from [32]); Table S4. Media recipes and antibiotics used in this study. [file 12915_2021_1142_MOESM1_ESM.pdf]

Competition-based screening helps to secure the evolutionary stability of a defensive microbiome: Additional file 1

Sarah F. Worsley[1], Tabitha M. Innocent[2,†], Neil A. Holmes[1,3,†], Mahmoud M. Al-Bassam[1], Morten Schiøtt[2], Barrie Wilkinson[3], J. Colin Murrell[4], Jacobus J. Boomsma[2*], Douglas W. Yu[1,5,6,*], Matthew I. Hutchings[1,3,*]

[1]School of Biological Sciences, University of East Anglia, Norwich Research Park, Norwich, Norfolk, UK, NR4 7TJ

[2]Centre for Social Evolution, Section for Ecology and Evolution, Department of Biology, University of Copenhagen, Copenhagen, Denmark.

[3]Department of Molecular Microbiology, John Innes Centre, Norwich Research Park, Norwich, Norfolk, UK, NR4 7UH

[4]School of Environmental Sciences, University of East Anglia, Norwich Research Park, Norwich, Norfolk, UK, NR4 7TJ

[5]State Key Laboratory of Genetic Resources and Evolution, Kunming Institute of Zoology, Chinese Academy of Sciences, Kunming, Yunnan, China 650223

[6]Center for Excellence in Animal Evolution and Genetics, Chinese Academy of Sciences, Kunming Yunnan, China 650223

†These authors contributed equally to the manuscript

*Correspondence: matt.hutchings@jic.ac.uk, jjboomsma@bio.ku.dk, douglas.yu@uea.ac.uk

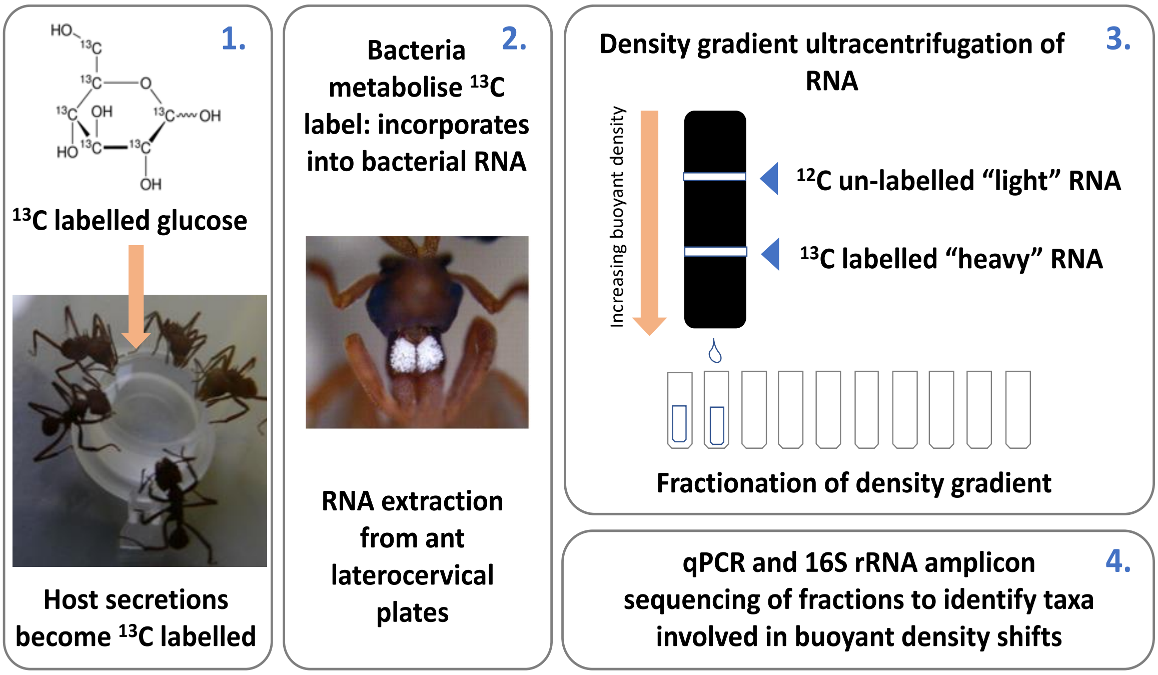

Figure S1. Overview of methodology used for RNA stable isotope probing of the propleural plate microbiome of *Acromyrmex echinatior* leaf-cutting ants.

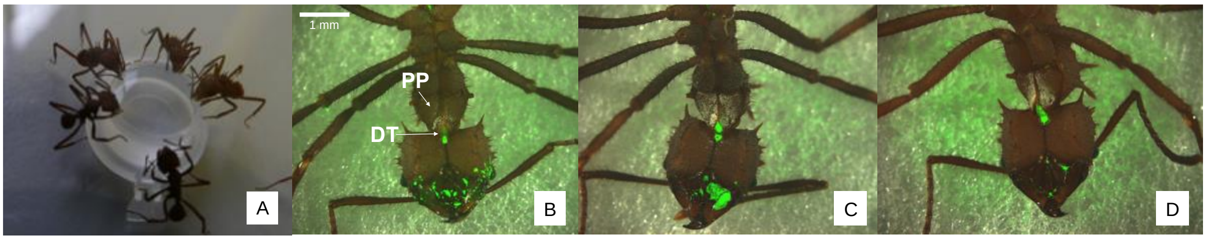

Figure S2. The extent to which a 20% (w/v) glucose water diet containing green fluorescent dye (A) was distributed over the ant body directly after taking a feed (B), 6 hours after feeding (C) and 24 hours after feeding (D). DT = fluorescence shining through from the ant digestive tract, PP = propleural plates with growth of actinobacteria.

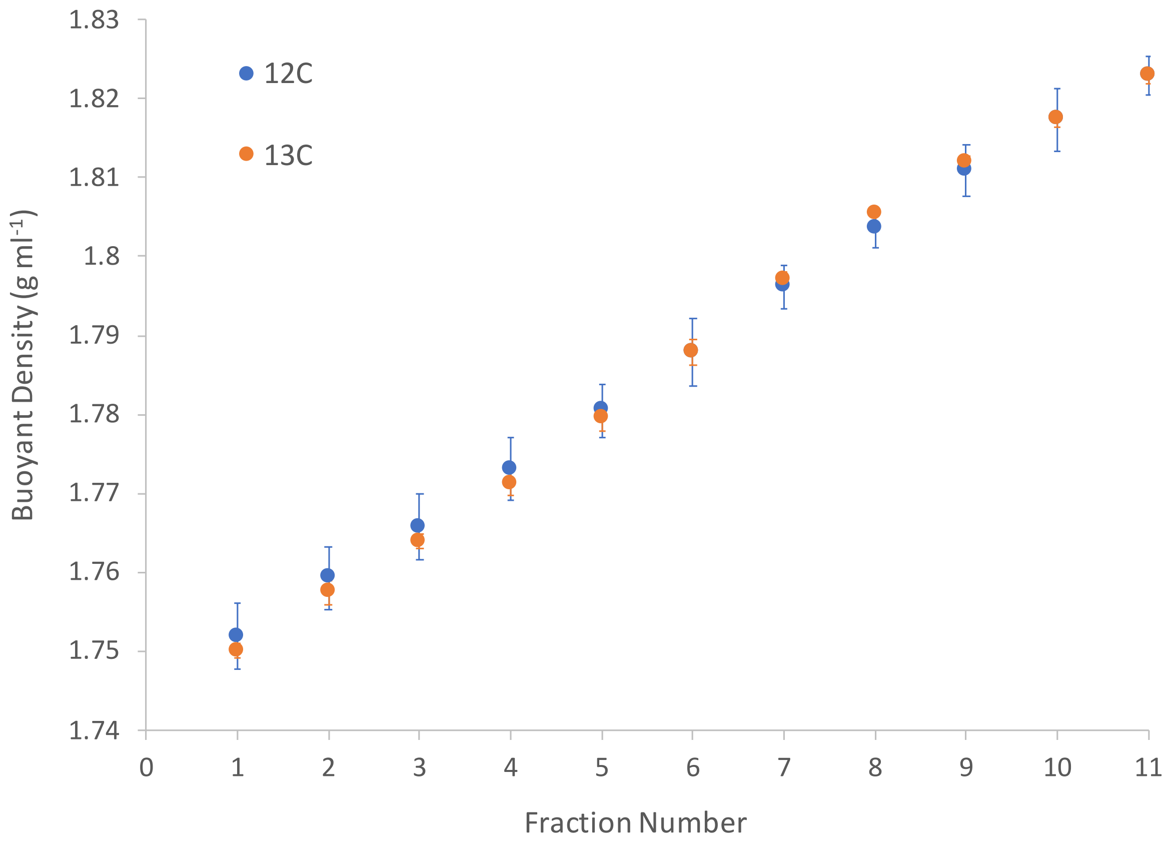

Figure S3. The relationship between RNA-SIP fraction number and buoyant density (in g ml^{-1}). Fractions were generated from density gradients containing RNA isolated from the propleural plates of *A. echinatior* ants fed on either a ^{12}C (blue) or a ^{13}C (orange) glucose water diet. Points represent averages (three samples each of 22 ants per dietary treatment) ± standard error.

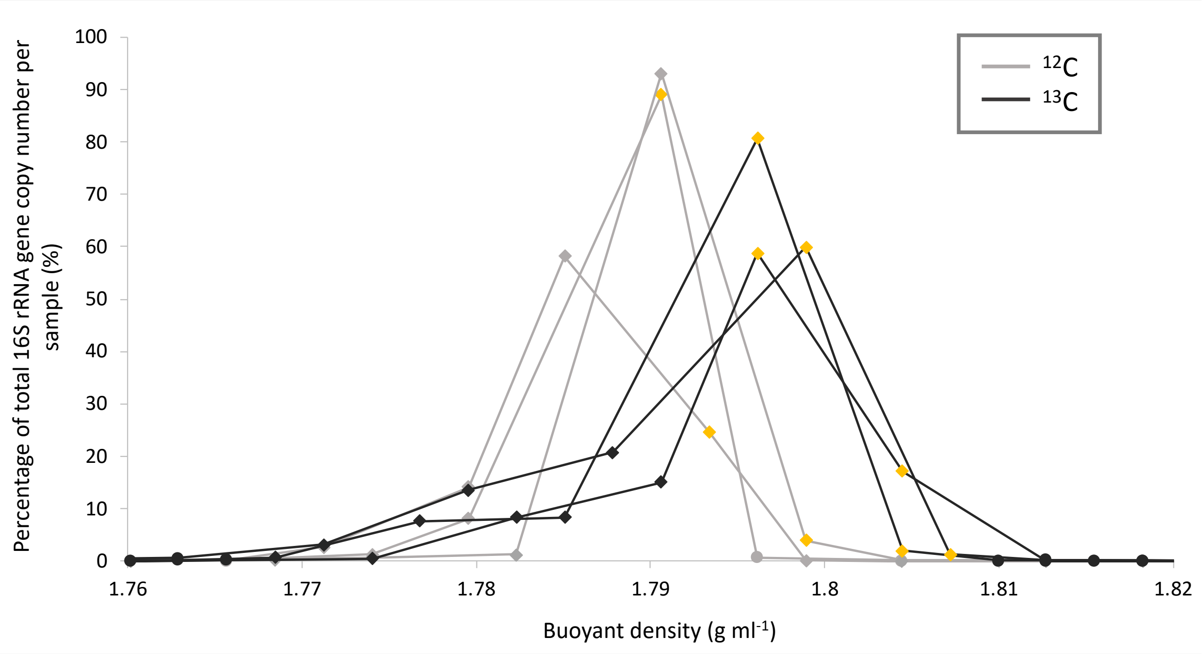

Figure S4. 16S rRNA gene copy number across different fractions of buoyant density gradients, as determined via qPCR. Gene copy number in each fraction is displayed as a percentage of total copy number in each sample. There were three replicate samples of 22 ants fed a ^{12}C (light grey) or ^{13}C (dark grey) glucose diet. Diamond symbols represent fractions that were sent for 16S rRNA gene amplicon sequencing and yellow diamonds represent those designated as “heavy” fractions under the different treatments.

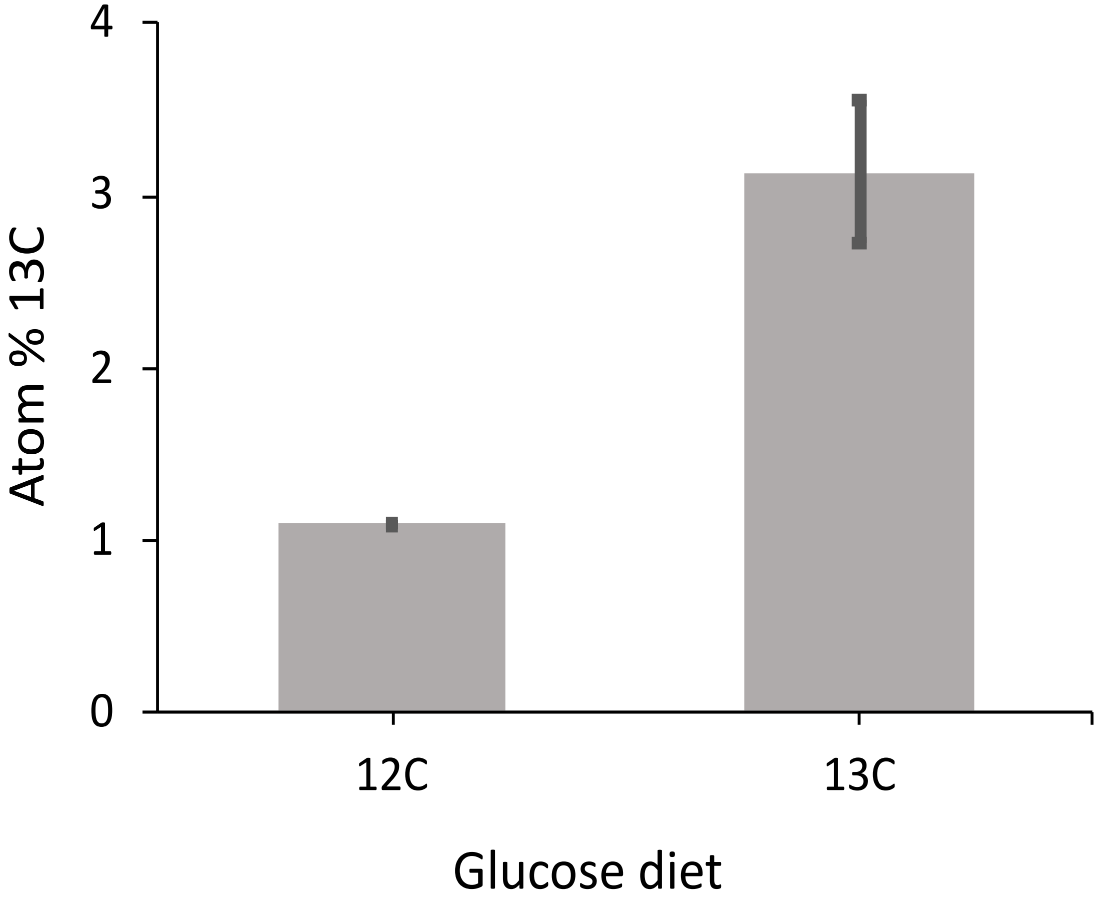

Figure S5. The atom percentage of ^{13}C in ants fed either a ^{12}C or ^{13}C labeled 20% (w/v) glucose diet for 10 days, as determined by Isotope Ratio Mass Spectrometry (IRMS) analysis.

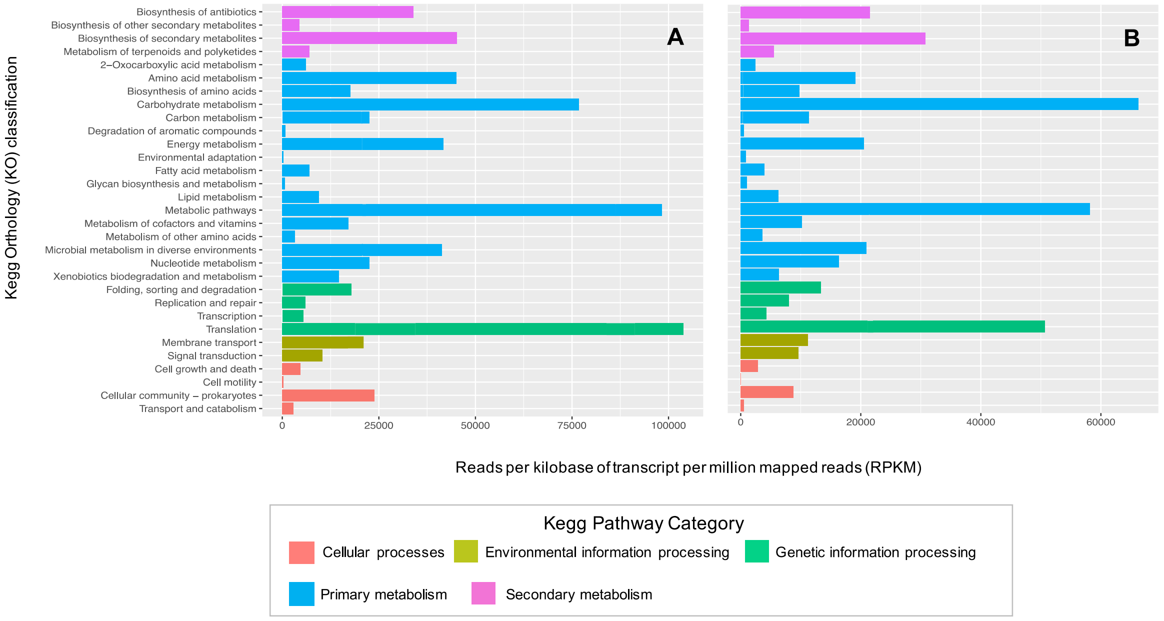

Figure S6. Expression levels (in reads per kilobase of transcript per million mapped reads, RPKM) of Kegg orthology pathway categories. (A) *Pseudonocardia octospinosus* (colony Ae088) and (B) *Pseudonocardia echinatior* (colony Ae1083) on the propleural plates of *Acromyrmex echinatior* ants. N= 1 sample of 80 pooled ants per colony.

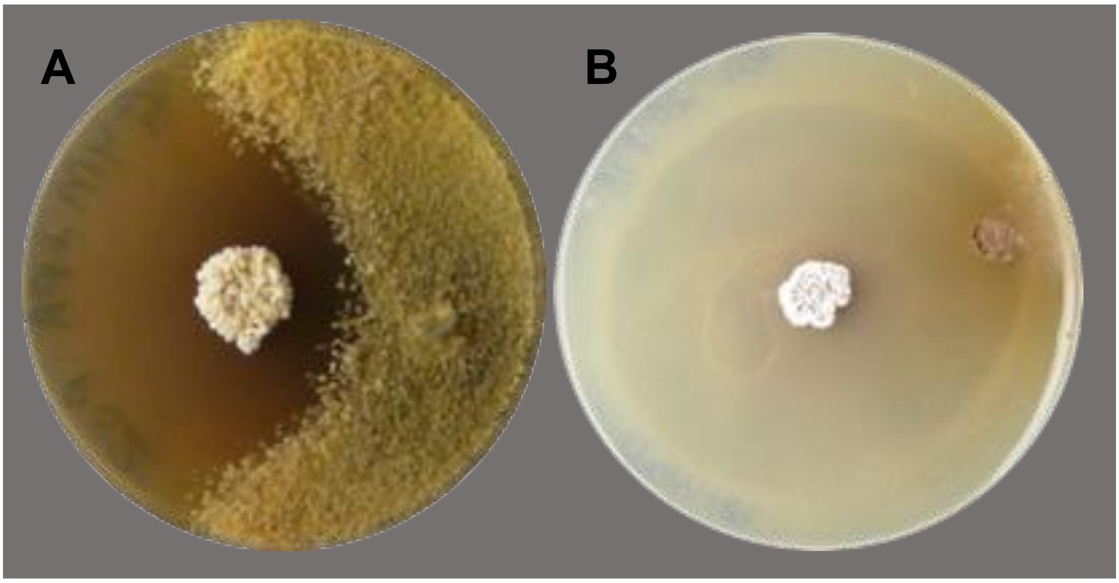

Figure S7. The bioactivity of *Pseudonocardia* isolates (**A**) *P. echinatior* PS088 and (**B**) *P. octospinosus* PS1083 against the specialized fungus-garden pathogen *Escovopsis weberi* (Table S1).

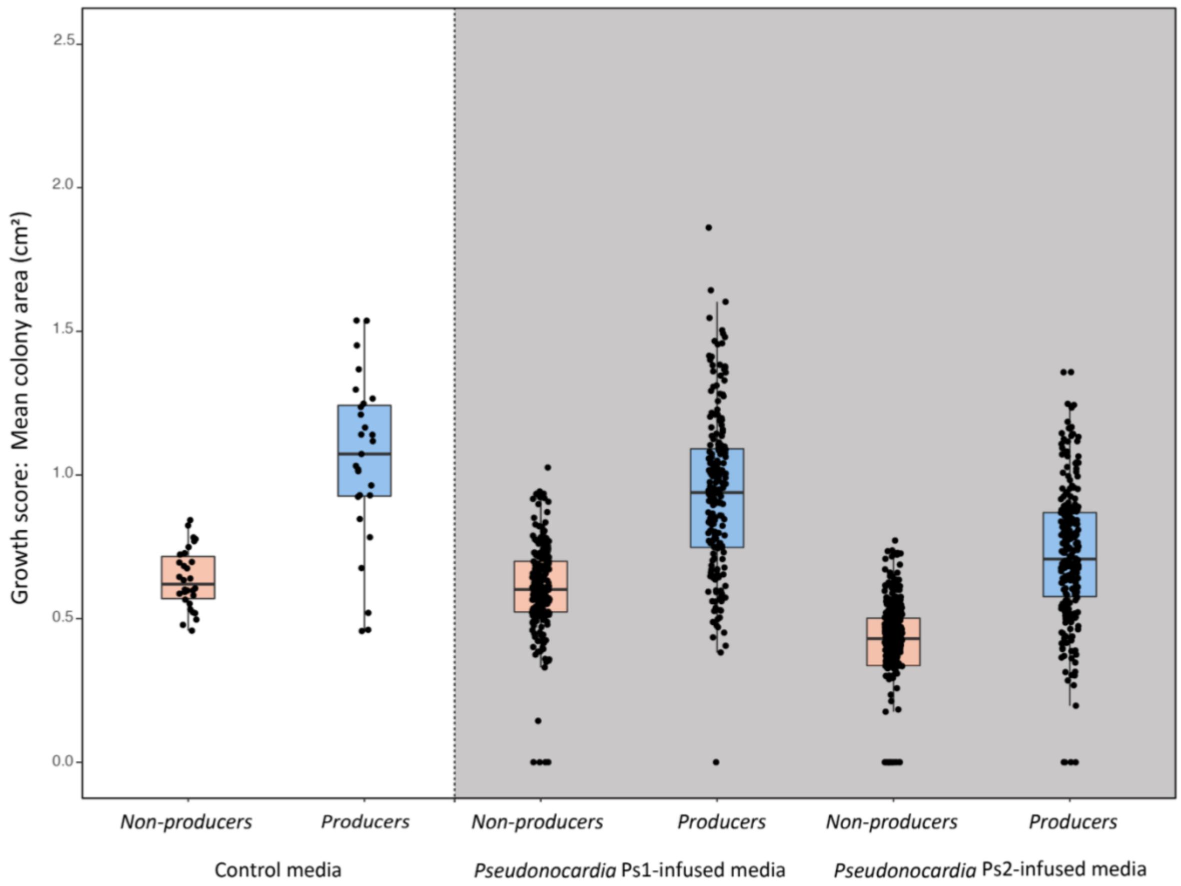

Figure S8. Individual growth-rate experiments of *Acromyrmex*-resident, non-producer strains, assessed as bacterial colony sizes after 5 days at 30 °C, with the boxplots indicating medians ± one quartile. The white section shows growth rates on control media, and the grey section shows growth rates on the *Pseudonocardia*-infused media. Red boxes represent non-producer strains, and blue boxes represent producer strains. For analysis, a linear mixed-effects model, including *Pseudonocardia* strain (n=17) and inoculated bacterial species (n=20) as random factors, was used to test for interactions and main effects of growth media (Control vs. Ps1-infused vs. Ps2-infused) and antibiotic production (non-producers vs. *Streptomyces*). There was no significant interaction effect ($\chi^2 = 2.64$, df = 2, p = 0.27), but both main effects were highly significant. The resident non-producers isolated from cuticular microbiomes had significantly slower growth rates on all media ($\chi^2 = 20.96$, df = 1, p < 0.0001) including the control media without antibiotics. This suggests that they are unable to outcompete producer strains on the cuticle of *Acromyrmex* ants and raises the question why these non-producer species can persist at all. Bacterial growth was also generally slower on Ps2-infused media than on Ps1-infused media ($\chi^2 = 21.43$, df = 1, p < 0.000 when analyzed in a balanced design without the control-media).

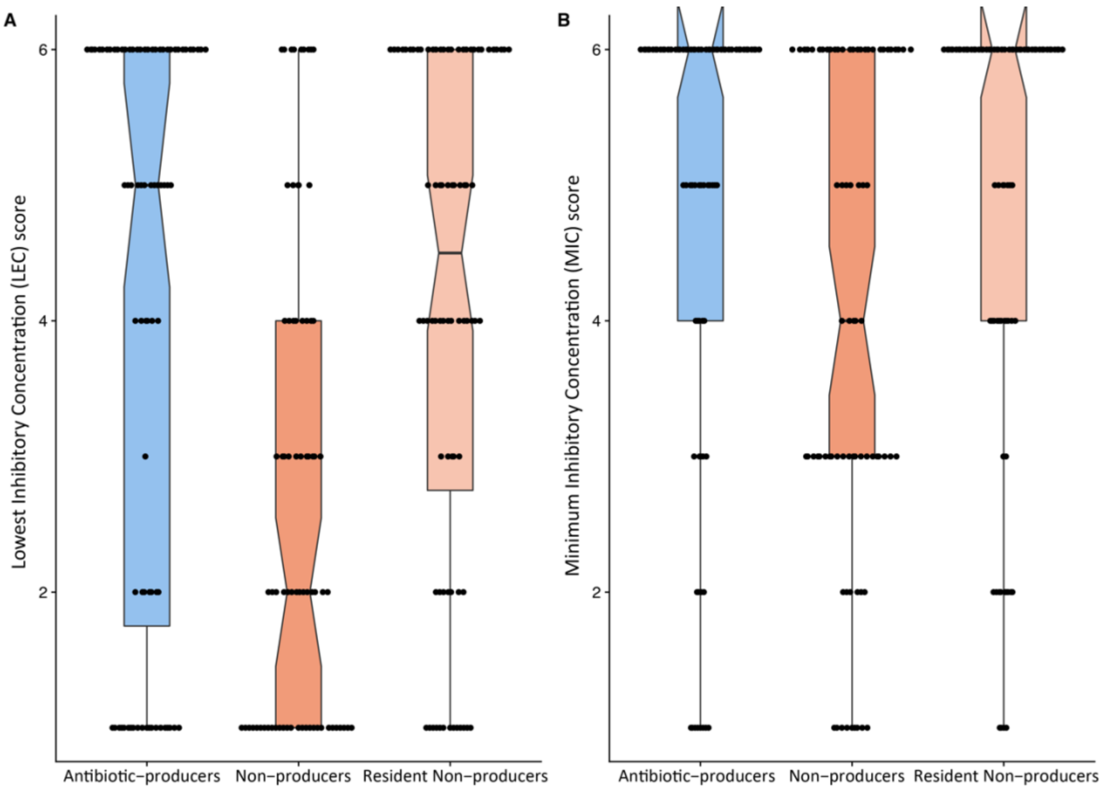

Figure S9. Antibiotic resistance profiles for producer, non-producer, and resident non-producer strains (Table S1). Boxplots indicate medians (notches) ± one quartile. For analysis, we calculated each strain's mean growth score across the eight tested antibiotics (reducing from n = 155 to n = 20). Producers showed higher levels of resistance than did non-producers for both measurements: Wilcoxon two-sided test (wilcox.test), W = 94.5, p = 0.0017 for LEC (**A**) and W = 80, p = 0.0253 for MIC (**B**), after correction for multiple testing. Producers and Resident non-producers showed no difference in resistance levels (p = 0.44 and 0.25). The 'rabbit ears' in **B** indicate that the medians are also the highest values. Data and details of the analysis are included in the code for Figure 5 (see Statistical Analyses in the Methods section).

Table S1. Details of ant colonies, bacterial strains and fungal strains, reference genomes and primers used in experiments. **Pseudonocardia* strains that have been genome-sequenced. †*Pseudonocardia* strains that were only used in the growth-rate experiment with non-producers.

**Ant colony name**	**Description**	**Origin**
Ae1083	*Acromyrmex echinatior* ant colony harboring *Pseudonocardia octospinosus*.	Gamboa, Panama.
Ae088	*Acromyrmex echinatior* ant colony harboring *Pseudonocardia echinatior*.	Gamboa, Panama.
**Microbial strain**	**description**	**origin**
PS1083	*Pseudonocardia octospinosus* isolated from *Acromyrmex echinatior* ants in colony Ae1083.	Isolated in this study
PS088	*Pseudonocardia echinatior* isolated from *Acromyrmex echinatior* ants in the colony Ae088.	Isolated in this study
CBS 810.71	Strain of the parasitic fungus *Escovopsis weberi.*	Westerdijk Fungal Biodiversity Institute
**8 *Pseudonocardia* Ps1 strains**		
Ae356*	Derived from colony Ae356	Holmes *et al.* [32]
Ae263*	Derived from colony Ae263	Holmes *et al.* [32]
Ae322	Derived from colony Ae322	This Study
Ae150A*	Derived from colony Ae150A	Holmes *et al.* [32]
Ae168*	Derived from colony Ae168	Holmes *et al.* [32]
Ae707-CP-A2* (Ae707_Ps1)	Derived from colony Ae707-CP-A2	Holmes *et al.* [32]
Ae712†	Derived from colony Ae712	This Study
Ae280†	Derived from colony Ae280	This Study

**11 *Pseudonocardia* Ps2 strains**		
Ae406*	Derived from colony Ae406	Holmes *et al.* [32]
Ae160	Derived from colony Ae160	This Study
Ae505*	Derived from colony Ae505	Holmes *et al.* [32]
Ae717*	Derived from colony Ae717	Holmes *et al.* [32]
Ae703	Derived from colony Ae703	This Study
Ae702	Derived from colony Ae702	This Study
Ae707	Derived from colony Ae707	This Study
Ae331*	Derived from colony Ae331	Holmes *et al.* [32]
Ae706*	Derived from colony Ae706	Holmes *et al.* [32]
Ae704	Derived from colony Ae704	This Study
Ae715	Derived from colony Ae715	This Study
**10 environmental antibiotic-producing strains (all *Streptomyces*)**		
S1. *S. coelicolor* M1146	*Streptomyce*s *coelicolor* M145 Δ*act* Δ*red* Δ*cpk* Δ*cda*	John Innes Centre, Norwich, NR4 7UH, UK [48]
S2. *S. lividans 66*	Soil derived *Streptomyce*s species	John Innes Centre, Norwich, NR4 7UH, UK [49]
S3. *S. coelicolor* M145	Soil derived *Streptomyce*s, SCP1- SCP2- Pgl+	John Innes Centre, Norwich, NR4 7UH, UK [50]
S4. *S. scabies* 87-22	Soil derived *Streptomyce*s species	Bignell *et al.* [51]
S5. *S. venezuelae* NRRL B-65442	Soil derived *Streptomyce*s species	USDA ARS Culture Collection. https://nrrl.ncaur.usda.gov/cgi-bin/usda Strain no. B-65442.
S6. *S.* Ae150A-B1	*Streptomyces* derived from lab workers of captive colony Ae150A	This study

S7. *S.* Ae356-S1	*Streptomyces* derived from lab workers of captive colony Ae356	This study
S8. *S. formicae* KY5	*Tetraponera penzigi* derived *Streptomyces* species	Seipke *et al.* [52], Holmes *et al* [53]
S9. *S.* S4	*Acromyrmex octospinosus* derived *Streptomyces albidoflavus* S4	Barke *et al.* [37]; Seipke *et al.*[47]
S10. *S.* S4 Δ*antA::apr*	*Streptomyces albidoflavus* S4 Δ*antA::apr*	Seipke *et al.* [54]
**10 environmental non-producer strains**		
St1. *Escherichia coli*	Non-pathogenic ESKAPE laboratory screening strain	ATCC® 11775™
St2. *Lysobacter antibioticus*	Non-pathogenic ESKAPE laboratory screening strain	Handelsman Lab, Small World Initiative, University of Wisconsin-Madison, USA
St3. *Bacillus subtilis*	Non-pathogenic ESKAPE laboratory screening strain	Handelsman Lab, Small World Initiative, University of Wisconsin-Madison, USA
St4. *Pseudomonas putida*	Non-pathogenic ESKAPE laboratory screening strain	Handelsman Lab, Small World Initiative, University of Wisconsin-Madison, USA
St5. *Erwinia caratova*	Non-pathogenic ESKAPE laboratory screening strain	Handelsman Lab, Small World Initiative, University of Wisconsin-Madison, USA
St6. *Enterobacter aerogenes*	Non-pathogenic ESKAPE laboratory screening strain	ATCC® 51697™
St7. *Acinetobacter baylyi*	Non-pathogenic ESKAPE laboratory screening strain	ATCC® 33305™

St8. *Staphylococcus epidermidis*	Non-pathogenic ESKAPE laboratory screening strain	ATCC® 14990™
St9. *Micrococcus luteus*	*Micrococcus luteus* (NCTC2665, “Fleming strain”)	ATCC® 4698™
St10. *Serratia* KY15	*Tetraponera penzigi* derived *Serratia*	Seipke *et al.* [52]
**10 *Acromyrmex*-resident non-producer strains**		
Sr1. *Ochrobactrum sp*	*Acromyrmex* derived	This study
Sr2. *Erwinia* sp.	*Isolated from large A. echinatior worker ants in lab colonies*	This study
Sr3. *Acinetobacter* sp.	*Isolated from large A. echinatior worker ants in lab colonies*	This study
Sr4. *Sphingobacterium sp.*	*Isolated from large A. echinatior worker ants in lab colonies*	This study
Sr5. *Acinetobacter* sp.	*Isolated from large A. echinatior worker ants in lab colonies*	This study
Sr6. *Luteibacter sp.*	*Isolated from large A. echinatior worker ants in lab colonies*	This study
Sr7. *Flavobacterium sp.*	*Isolated from large A. echinatior worker ants in lab colonies*	This study
Sr8. *Brevundimonas sp.*	*Isolated from large A. echinatior worker ants in lab colonies*	This study
Sr9. *Acinetobacter sp.*	*Isolated from large A. echinatior worker ants in lab colonies*	This study

Sr10. *Brachybacterium sp.*	*Isolated from large A. echinatior worker ants in lab colonies*	This study
**Genome**	**Description**	**Accession number/ reference**
Acromyrmex echinatior	Whole genome shotgun sequencing project of the *A. echinatior* genome.	AEVX00000000; Nygaard *et al.* [55]
Ae707	Whole genome shotgun sequencing of a wild-type isolate of *Pseudonocardia octospinosus*, isolated from the cuticle of a large worker.	MCIR00000000; Holmes *et al.* [32]
Ae706	Whole genome shotgun sequencing of a wild-type isolate of *Pseudonocardia echinatior*, isolated from the cuticle of a large worker.	MCIQ00000000; Holmes *et al.* [32]
**Primer**	**Sequence**	**Reference**
341F	5'-CCTACGGG AGGCAGCAG-3'	Amplifies the V3 region of the 16S rRNA gene [56]
518R	5'- ATTACCGCGGCTGCTGG -3'	

Table S2. The total number of RNA-sequencing reads (and percentage of total reads in brackets) originating from propleural plate samples taken from the ant colonies Ae1083 or Ae088, respectively, that successfully aligned to the *A. echinatior* genome (Table S1) and to the genomes of the *Pseudonocardia* species associated with the ant colony of origin (*P. octospinosus* or *P. echinatior*, respectively).

**Sample**	**Source**	**Alignment**		
		***A. echinatior* genome**	***P. octospinosus* genome Ae707**	***P. echinatior* genome Ae706**
Ae1083	80 pooled sets of propleural plates from large workers	8,548,640 (78.7 %)	103,820 (1.0 %)	-
Ae088	80 pooled sets of propleural plates from large workers	7,058,678 (73.5 %)	-	189,989 (2.0 %)

Table S3. Secondary metabolite BGCs in the *Pseudonocardia* mutualist genomes (table adapted from Holmes *et al.* [32]) and their associated expression values (in reads per kilobase of transcript per million mapped reads, RPKM) in RNA-sequencing experiments. Yellow rows are BGCs shared between strains. Green and blue represent BGCs that are unique to *P. octospinosus* and *P. echinatior*, respectively.

Cluster number		Classification	Cluster code	RPKM expression values	
P. octospinosus	*P. echinatior*			*P. octospinosus*	*P. echinatior*
1	7	Oligosaccharide	A	57.05	45.47
5	5	Terpene	B	81.19	37.58
7	4	Nystatin	C	1.76	24.28
8	2	Terpene	D	341.82	424.23
11	9	Bacteriocin	E	118.95	80.24
14	8	Ectoine	F	453.11	192.08
2	-	Other	G	34.34	-
3	-	Other	H	110.18	-
4	-	NRPS	I	10.97	-
6	-	Bacteriocin	J	111.07	-
9	-	NRPS	K	41.92	-
12	-	Other	L	52.55	-
13	-	NRPS	M	25.64	-
-	1	Other	N	-	16.75
-	3	T1PKS-NRPS	O	-	16.87
-	6	Bacteriocin	P	-	91.71
-	10	Terpene	Q	-	23.75
-	11	Bacteriocin	R	-	30.50

Table S4. Media recipes and antibiotics used in this study.

Medium Name	Component	g L^{-1} dH_2O	
Soya Flour Mannitol (SFM) Agar	Soy flour<br>Mannitol<br>Agar	20<br>20<br>20	
Potato Glucose Agar (PGA)	PGA (Sigma Aldrich)	39	
Glucose, Yeast, Malt (GYM) Agar	Glucose<br>Yeast extract<br>Malt extract<br>CaCO3<br>Agar	4<br>4<br>10<br>2<br>15	
Lennox Broth (LB)	Tryptone<br>NaCl<br>Yeast extract	10<br>10<br>5	
**Antibiotic**	**Concentrations Used**	**Target**	**Compound Type**
Chloramphenicol	0, 2.5, 5, 10, 25, 50 µg/ml	Translation inhibitor	Synthetic
Rifampicin	0, 0.5, 1, 2, 5, 10 µg/ml	Translation inhibitor	Polyketide
Streptomycin	0, 5, 10, 20, 50, 100 µg/ml	Translation inhibitor	Aminoglycoside
Vancomycin	0, 1, 2, 4, 10, 20 µg/ml	Cell wall synthesis inhibitor	Glycopeptide

Phosphomycin	0, 5, 10, 20, 50, 100 µg/ml	Cell wall synthesis inhibitor	Small molecule
Nalidixic Acid	0, 2.5, 5, 10, 25, 50 µg/ml	DNA gyrase inhibitor	Synthetic quinolone
Apramycin	0, 5, 10, 20, 50, 100 µg/ml	Translation inhibitor	Aminoglycoside
Ampicillin	0, 10, 20, 40, 100, 200 µg/ml	Cell wall synthesis inhibitor	ß lactam
